# First-Passage Time Analysis Based on GPS Data Offers a New Approach to Estimate Restricted Zones for the Management of Infectious Diseases in Wildlife: A Case Study Using the Example of African Swine Fever

**DOI:** 10.1155/2023/4024083

**Published:** 2023-08-25

**Authors:** Elodie Wielgus, Alisa Klamm, Franz J. Conraths, Carsten F. Dormann, Maik Henrich, Franz Kronthaler, Marco Heurich

**Affiliations:** ^1^National Park Monitoring and Animal Management, Bavarian Forest National Park, Freyunger Straße. 2, Grafenau 94481, Germany; ^2^Hainich National Park Administration, Department of Conservation and Research, Bei der Marktkirche 9, Bad Langensalza 99947, Germany; ^3^Friedrich-Loeffler-Institut, Federal Research Institute for Animal Health, Institute of Epidemiology, Südufer 10, Greifswald-Insel Riems 17493, Germany; ^4^Faculty of Environment and Natural Resources, University of Freiburg, Tennenbacher Straße. 4, 79106, Freiburg, Germany; ^5^Chair of Wildlife Ecology and Management, Faculty of Environment and Natural Resources, University of Freiburg, Tennenbacher Straße 4, 79106, Freiburg, Germany; ^6^Bavarian Health and Food Safety Authority, Veterinärstraße 2, Oberschleißheim 85764, Germany; ^7^Institute of Forestry and Wildlife Management, Inland Norway University of Applied Sciences, NO-2480 Koppang, Koppang, Norway

## Abstract

An essential part of any disease containment and eradication policy is the implementation of restricted zones, but determining the appropriate size of these zones can be challenging for managers. We designed a new method, based on animal movement, to help assess how large restricted zones should be after a spontaneous outbreak to successfully control infectious diseases in wildlife. Our approach uses first-passage time (FPT) analysis and Cox proportional hazard (CPH) models to calculate and compare the risk of an animal leaving different-sized areas. We illustrate our approach using the example of the African swine fever (ASF) virus and its wild pig reservoir host species, the wild boar (*Sus scrofa*), and we investigate the feasibility of applying this method to other systems. Using GPS data from 57 wild boar living in the Hainich National Park, Germany, we calculate the time spent by each individual in areas of different sizes using FPT analysis. We apply CPH models on the derived data to compare the risk of leaving areas of different sizes and to assess the effects of season and the sex of the wild boar on the risk of leaving. We conduct survival analyses to estimate the risk of leaving an area over time. Our results indicate that the risk of leaving an area decreases exponentially by 10% for each 100 m increase in radius size so that the differences were more pronounced for small sizes. Furthermore, the probability of leaving increases exponentially with time. Wild boar had a similar risk of leaving an area of a given size throughout the year, except in spring and winter, when females had a much lower risk of leaving. Our findings are in agreement with the literature on wild boar movement, further validating our method, and repeated analyses with location data resampled at different rates gave similar results. Our results may be applicable only to our study area, but they demonstrate the applicability of the proposed method to any ecosystem where wild boar populations are likely to be infected with ASF and where restricted zones should be established accordingly. The outlined approach relies solely on the analysis of movement data and provides a useful tool to determine the optimal size of restricted zones. It can also be applied to future outbreaks of other diseases.

## 1. Introduction

Wildlife diseases may cause substantial suffering and losses in the affected animal species, in addition to becoming a serious threat if the pathogens are transmitted from wildlife to livestock [1] or, in the case of zoonoses, to humans [2]. They can also endanger the conservation of biodiversity [3]. Increasing and expanding human populations and the associated land-use changes (habitat fragmentation), together with climate change, have exacerbated the risk of disease transmission between and within wild and domestic animal populations [4, 5]. The prevention and control of infectious diseases in wildlife populations have thus become challenging tasks for ecologists, wildlife managers, and veterinary authorities since they are connected to broader issues related to human health, livestock economics, animal welfare, and food safety.

An important part of many wildlife disease control policies is establishing “restricted zones” (as defined by the Commission Delegated Regulation (EU) 2020/687, i.e., the “Animal Health Law”) for disease control or surveillance immediately after the first disease case has been detected in a new territory; for example, to control and monitor bovine tuberculosis and chronic wasting disease in wild deer ([6, 7], respectively), bovine tuberculosis in possums [8], African swine fever (ASF) in wild boar [9] and rabies in raccoons [10]. This approach aims to reduce the disease prevalence in affected areas and to avoid spatial spread by implementing a combination of measures and restrictions in each zone. Measures include, but are not limited to, the building of fences to delineate the affected area, prevent animal movement between affected and disease-free areas, help to reduce the contact rates among individuals and groups by creating a habitat fragmentation effect, and establish the epidemiological situation of the population in that area (e.g., the number of animals testing positive or negative for virus genes or antibodies against the virus); vaccination, if available; carcass removal; and reduction of population size [10–12]; hunting, agriculture, and tourism may also be limited to avoid animal disturbance and human-mediated spread of the disease. Restricted zones are usually designed by assuming that most host animal movements are contained therein, while taking into account the human, logistical and financial resources available. The size of these zones is therefore critical. A zone that is too large will compromise the effectiveness of the mitigation measures (i.e., animal removal may become too labor-intensive for a sufficient number of infected animals to be eliminated) and adversely impact stakeholders, such as farmers, foresters, and hunters. Conversely, a zone that is too small will increase the risk that infected animals leave or have already left the area and spread the disease elsewhere. There is no formal consensus on the appropriate size, or even shape, of such zones, but these characteristics must be based on the movement and space-use patterns of the disease-spreading species, which can be highly variable depending on habitat type, landscape structure and configuration. This may result in a fixed radius to be applied around the locations where positive disease cases have been detected [7, 10, 13]. Knowledge of host species movement patterns is therefore essential and requires detailed data on animal movements [12].

During the last decades, our knowledge on the movement patterns of free-ranging animals has greatly improved following advances in telemetry [14] and the development of the corresponding statistical methods [15]. One such method is the first-passage time (FPT), defined as the time required for an animal to cross a circle of a given radius around its present location [16]. The FPT has been used extensively in foraging ecology and habitat-use studies to determine where animals concentrate their search efforts along movement paths [16–18]. Recent developments in the FPT framework have further expanded the scope of its use. For example, FPT has been combined with segmentation processes to describe and detect changes in migration movements [19] and with survival models in studies of habitat selection [20, 21]. To infer habitat selection, Freitas et al. [21] proposed an objective method to estimate the risk of an animal leaving an area of a given radius by treating FPTs as survival data.

Here, we adapt the approach of Freitas et al. [21] to develop a new, powerful movement ecology-based tool to aid in the control of infectious diseases in wildlife. Using location data, our method quantifies the risk of an animal leaving areas of different sizes to guide the establishment of appropriately sized restricted zones, in particular, the buffer around the outbreak site, with animal movements as a key risk factor for disease spread. We illustrate the applicability and strength of this approach using a case study of ASF virus and its wild pig reservoir host species, the wild boar (*Sus scrofa*) and we investigate the potential suitability of this method for broad application. ASF has become a top-priority animal disease in the European Union. Since the introduction of genotype II into Georgia in 2007, it spread across Europe and was transmitted to China and reached several Asian countries. It has also been recently detected in the Dominican Republic and Haiti, thus threatening the Americas [9]. In wild boar populations in Eastern and Central Europe, the disease has become established in self-sustaining infection cycles [22]. Infections of wild boar and domestic pigs (*S. scrofa domesticus*) with the highly virulent variants of genotype II cause high case/fatality ratios of almost 100%. After an incubation period of 3–5 days, infected wild boar develop clinical signs, such as high fever, anorexia and reduced mobility, and usually die within ∼2 weeks postinfection [23–26]. Recent reports suggested that virus strains with reduced virulence or attenuation and lower mortality rates might also circulate in Europe [27–30]. The main route of disease transmission between wild boar is direct contact with infected individuals or to carcasses of wild boar that succumbed to ASF, but the ASF virus can remain infectious in the environment (e.g., soil or water) for several weeks or months, and indirect transmission through fomites and contaminated meat may play an important role, in particular in transmitting ASF over long distances [22, 31, 32]. Outbreaks may cause significant economic losses to the pig industry due to trade restrictions, or culling, if the disease is transmitted to domestic pigs.

According to recommendations of the World Organisation for Animal Health (WOAH, formerly known as OIE), restricted zones should be established as follows in the case of a focal introduction of ASF into a wild boar population: A fenced “infected zone,” where the virus is actively circulating, is surrounded by a “buffer zone” where disturbance of wild boar should be kept minimal, for example, by feeding and hunting bans, but where restrictions for wild boar, pigs and products derived from these animals are high. In the infected and buffer zones, the search for carcasses of infected animals, their removal and safe disposal must be intensified. In the periphery, “control zones” should be established, in which wild boar densities should be reduced as much as possible using different measures (intensive hunting, culling, or trapping), but still with minimal disturbance [9, 13, 33, 34]. The infected zone is defined as the area including all reported ASF cases in wild boar and a buffer area (thereafter called “buffer”) of a certain distance around the cases, where infected individuals are already expected to be found due to their movement. During the ASF epidemic in the Czech Republic in 2017, the infected zone and fence placement have been determined based on the average annual home range of a wild boar and the expected speed of the epidemic wave [34], but, in general, strategies to adequately quantify wild boar movement and precise recommendations are missing. Quantifying the risk of leaving an area of a given radius allows evaluating how far an individual that has been in contact with the carcass of an ASF-infected wild boar could have moved in a certain time window, and thus implementing the infected zone accordingly and possible barrier placement. By quantifying this risk, it is possible to estimate the benefits (e.g., economically) of reducing restricted zones against the costs of the disease spreading faster and further. In addition, it can provide conservative estimates of the size of the area that a dead ASF-infected wild boar might have used a few days before its death while it was transmitting the virus.

## 2. Materials and Methods

### 2.1. Study Area

**Figure 1:**
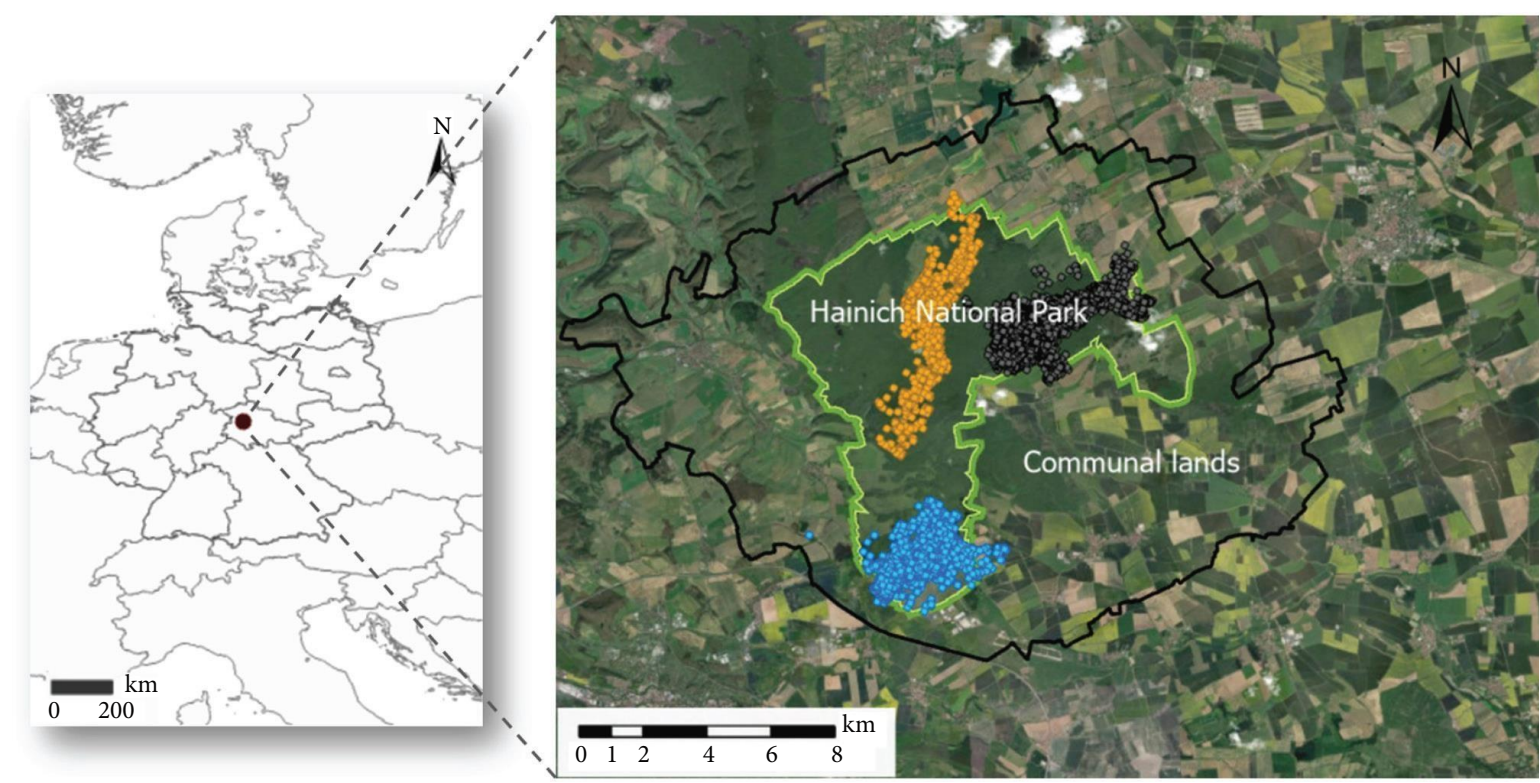
Location of the study area, in Thuringia, Germany, was calculated as the MCP95 of all wild boar locations considering districts (black line in the inset). The GPS-tracked wild boar occupied the Hainich National Park and its surroundings. The trajectories of three individuals (orange: juvenile female, blue: subadult male, gray: adult male) are shown as an example.

The study was conducted in Hainich National Park (HNP) and its surroundings in Thuringia, Central Germany (∼25,000 ha, 51° 06′ N, 10° 52′ E, Figure 1) from October 1, 2016 through December 31, 2019. The study area (HNP and surrounding areas) consisted of 54.6% agricultural lands (crops, arable lands, and pastures), 34.8% forests, 7.3% open lands (natural grasslands and transitional woodland shrubs), 3.2% anthropogenic areas (urban fabric, industrial, commercial and transport units) and 0.4% water bodies (based on Corine Land Cover database, European Environment Agency, http://www.eea.europa.eu/). The altitude of the study area ranges between 180 and 350 m a.s.l. The HNP is about 75 km^2^ large in size, and part of the HNP (approximately 20%) is within the boundaries of a UNESCO World Heritage Site (“Ancient and Primeval Beech Forests of the Carpathians and Other Regions of Europe”), where any form of hunting is strictly prohibited. Over the years, this zone has increased due to advancing succession and the associated restrictions on hunting activities; in addition, a partial area cannot be hunted due to the presence of old military sites. The total area of this “no-hunting zone” is about 33 km^2^. During the project period, wild boar hunting was allowed in the rest of the park from the beginning of September to mid-January. Hunting on the remaining areas in the study area is based on the hunting seasons of the state of Thuringia. In 2018, the population density of wild boar in the HNP was estimated to be 11.7 (8.7–15.7) animals/km^2^ [35]. In addition to wild boar, the most common herbivore species are roe deer (*Capreolus capreolus*), fallow deer (*Dama dama*), and red deer (*Cervus elaphus*).

### 2.2. Movement Data

We used GPS data of 57 ASF-free wild boar collected as part of a space-use study [35]. We excluded six individuals from the original study due to the short tracking period (<10 days). The handling procedure was approved by the regional Veterinary Authority (Free State of Thuringia) and fulfilled the requirements of animal welfare (permit no. 15-109.16). Wild boar were captured at four different locations in the HNP, each in the close proximity of the no-hunting zone. Wild boar were trapped in wood-clad corral traps of ∼30 m^2^ equipped with live cameras for monitoring and with remote-controlled gates. We drove a caught wild boar into a net tunnel, and two or three people held it on the ground with its eyes covered with a cloth. Each boar was fitted with a Vectronic Aerospace GPS-GSM Vertex Lite collar. The weight of the collared animals was 30–80 kg. When several individuals were simultaneously caught in the trap, we marked as many wild boar as possible with GPS collars, if the size of the individuals allowed doing so. The collaring operation took 3–5 min per animal, after which the animal was released at the capture site. Further details on GPS deployment and monitoring can be found in Klamm et al. [35].

The animals were classified according to age and sex at collaring by experienced wildlife biologists and managers based on size and coat color. Animals were considered juveniles at an age between 6 and 12 months, yearlings at an age between 1 and 2 years, and adults at >2 years. GPS collars were deployed on 3 juvenile females, 19 yearling females, 7 adult females, 5 juvenile males, 21 yearling males, and 2 adult males. Of these, 45 individuals belonged to 16 different groups. The number of collared individuals in the same group varied between two and seven. The remaining 12 individuals were either solitary or members of unmarked groups [35].

GPS loggers were programed to record a location every 30 min (48 times a day). We omitted locations recorded during the first week of postcapture monitoring and collaring to remove potential bias introduced by the initial capture and handling [36, 37]. The resulting duration of tracking varied between 10 days and 437 days (mean = 142 days, median = 109 days). We projected the coordinates of locations into the WGS84/UTM zone 32 N.

### 2.3. FPT Analysis

The time spent by a wild boar in areas of different sizes was calculated by FPT analysis [16]. As the FPT method is most effective when the data are collected at regular time intervals [16], we removed possible gaps in the location data of each individual by dividing the path into several segments when the time between successive locations was >3 hr (i.e., all successive locations with an interval >3 hr were excluded from the analyses). We provide a quantitative assessment of the effects of acquisition rate and the cutoff value for separating the paths on the further analyses in the Supporting Information (Supplementary 1) to assess the generality of our approach. Because the accuracy of FPT analysis depends on the tracking duration [18], tracking records that did not include at least 25 consecutive locations (>12 hr in duration) were removed from the analyses.

For each segment of an individual's path, we calculated the time spent in circles centered around each location of that individual. When the first and/or last crossing of the circle was unknown, we used the first and/or last known location in the circle to calculate the minimum time spent in the circle instead. This typically occurred at the beginning or end of a track of a boar that never moved outside the circle. We calculated the time/minimum time spent by an individual in circles with radii ranging from *r* = 1 to 20 km, at 1 km increments from 1 to 10 and 5 km increments thereafter. We chose *r* following the Commission Delegated Regulation (EU) 2020/687 for ASF outbreaks in domestic pigs, which requires the establishment of a protection zone (PZ) and a surveillance zone (SZ) with *r* > 3 and *r* > 10 km, respectively, around the presumed starting point of the infection, i.e., the geographic position of the first detected (index) case.

### 2.4. Statistical Analysis

We fit Cox proportional hazard (CPH) models according to Freitas et al. [21] to (1) test the influence of the radius size on the period spent by a collared wild boar inside the circle and (2) to calculate the instantaneous probability (risk) that a boar would leave a circle with a given radius, using survival functions. The time spent in the circles, representing the time to the event (leaving a circle of radius *r*), was treated as survival time (the response variable in CPH models). When we calculated a minimum time spent in the circle (when the positions entering and leaving the circle were unknown), we treated these times as right-censored survival times (i.e., less weighting) since the duration was smaller than expected compared to a situation where the positions entering and leaving the circle are known. To investigate whether the risk of leaving varied across seasons, we split the data into seasons. Seasons were defined as winter (December–February), spring (March–May), summer (June–August), and autumn (September–November) to reflect changes in climate, vegetation, and the life cycle of wild boar. Since previous studies reported differences in the spatial behavior of males and females [38], we also tested whether the risk of leaving differs by sex. We included the following fixed effects in the CPH model: season and sex (categorical) and *r* (numerical). We considered all possible two-way interactions between explanatory variables. To account for repeated, nonindependent observations from the same individual, we ran one CPH model with individual identity as a random effect, using the “coxme” package (Therneau 2020). Survival functions S(*t*) that use random-effects CPH models to predict the probability of an animal staying in an area longer than a time *t* have not yet been implemented in the available software. Instead, a fixed-effects CPH model (i.e., no random effects) was additionally fitted using the R package “survival” [39]. An alternative CPH model with equally weighted individuals, i.e., the weights of individuals with *k* events were reduced by assigning them a weight of 1/*k* for each data point, is shown in Supplementary Information. The different approaches give slightly different quantitative results, but the magnitude is small, and it is unlikely to change the qualitative conclusions of our study (Supplementary 1).

The hazard function for the *i*th individual in the *j*th season and the *r*th radius size, that is, the risk that an animal leaves an area of radius *r* at time *t* during season *j*, is defined as follows:

(1) $$\begin{array}{c} h_{i}\left(t\right)=\exp\left(\beta_{r}\text{radius}+\beta_{i}{\text{sex}}_{i}+\beta_{j}{\text{season}}_{j}+\beta_{ir}{\text{sex}}_{i}\text{radius}+\beta_{jr}{\text{season}}_{j}\text{radius}+\beta_{ij}{\text{sex}}_{i}{\text{season}}_{j}+b_{i}\right)\;h_{0}\left(t\right), \end{array}$$

where *radius* is the radius size of the circle (in meters), sex_*i*_ is the sex of the animal in the *i* category, season_*j*_ is the season of the year in the *j* category, *b*_*i*_ is the per-individual random effect and *h*_0_(*t*) is the baseline hazard function at time *t* (the risk of leaving an area where all explanatory variables are equal to zero or to the reference level).

We calculated hazard ratios (HRs) from the exponential of each coefficient to quantify the effects of the radius *r* of an area, the season, and sex on the risk of leaving an area [21, 40]. An HR >1 indicates an increased risk of leaving, while an HR <1 indicates a lower risk of leaving. Thus, for *r*, which is a continuous variable, an HR equal to 1.4, for example, indicated that the risk of leaving increased by 1.4 times (40%) for each 1 m increase in *r*, and an HR less than one is interpreted in the opposite way. In the same way, for sex and season, which are categorical variables with ≥2 levels, the first level (male and autumn) was considered the reference level; for any other level, an HR equal to 0.8, for example, indicated that the risk of leaving decreased by 0.8 times (20% less) compared to the reference level. We do not use *p*-values as they could be misleading given our large sample size [41]. Survival functions calculated from the fixed-effects CPH model were used to derive the risk of leaving an area over time for each sex, season, and *r*. All analyses were performed in the R statistical computing environment [42].

## 3. Results

We used trajectories from 57 wild boar representing 375,729 locations and 842 days of tracking data. Due to data selection and processing (see Section 2), we excluded ∼7% of the total records of our initial GPS data. Although only 14 individuals provided data for analysis throughout the year (i.e., all four seasons), we had data of 11 and 18 individuals for three and two seasons, respectively. In total, for each season, we had data from at least 28 individuals (max = 40). Mean (±SD) interval between successive locations was 32.4 ± 27.2 min, and the mean and median duration of tracking records were 209.9 and 41.0 hr, respectively.

**Figure 2:**
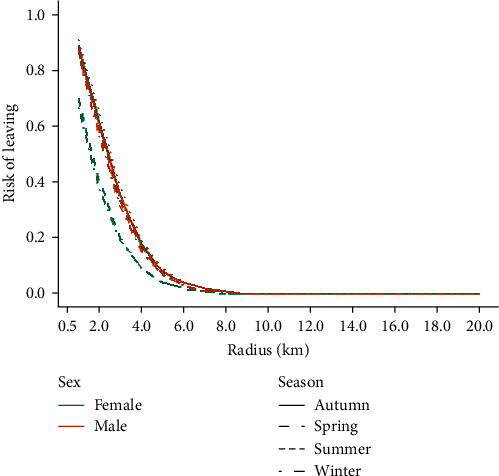
Relationship between the predicted risk of leaving and the radius size at *t* = 14 days, depending on the season and sex. The predictions were based on the fixed-effects CPH model.

**Figure 3:**
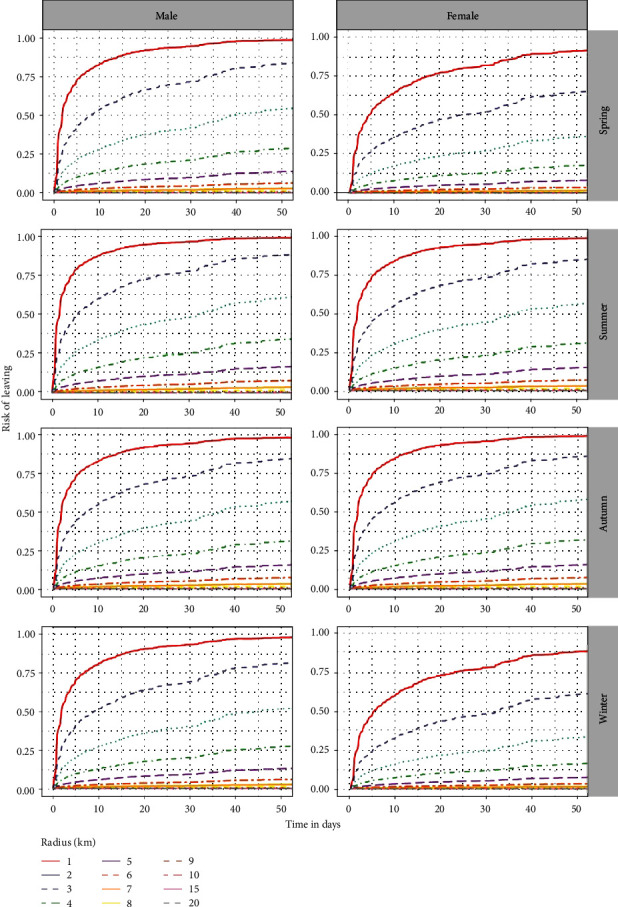
Predicted risk of a wild boar leaving a circle with a radius of 1–20 km after a given period (in days), depending on the season and sex. The predictions were based on the fixed-effects CPH model.

Based on the estimated HRs (Table 1), the risk of leaving an area decreased with *r* in all seasons and for both males and females. For each 1 m increase in radius size, the risk of leaving decreased exponentially by about 0.999 times (i.e., at a rate of 10% 100 m^−1^, Figure 2). The effect of sex on the risk of leaving was dependent upon the season and vice versa. In general, females had a lower risk of leaving an area of any radius size than males (0.5–0.8 times lower), indicating that females were more likely to remain in the same area for a given amount of time compared to males. However, in autumn, the risk of leaving an area was similar for females and males (only ∼1.1 times higher for females than for males, Table 1 and Figure 3). There were few differences in the risk of leaving an area of any radius size for males between seasons (∼0.9–1.1 times the risk of leaving in autumn, Table 1 and Figure 3). In contrast, for females, the risk of leaving was considerably lower in winter and spring compared to autumn (∼0.5 times lower than the risk in autumn). In general, there was good consistency between *β* coefficients from the fixed- and random-effects CPH models (Table 1). The biggest difference between the model types concerned the effect of season, where the difference in HRs can be much as 0.2 (Table 1). Predictions of the risk of leaving an area at different times and the time when the risk of leaving reaches specific values for each radius size, sex, and season are provided in the Supplementary Information (Supplementary 1).

The risk of leaving increased exponentially with time, independent of season, and sex (Figure 3). Although the risk of leaving an area decreased with *r*, the differences in the risk of leaving were more pronounced for *r* < 6 km as time goes (Figures 2 and 3). In general, for *r* ≥ 6 km, the risk of leaving for a given period of time decreased only slightly with increasing *r*.

The average risk of leaving refers to a male wild boar in autumn for an average radius size (*r* = 7.5 km). The variance component attributed to individual variability (*b* in the hazard function above) was 1.047. The standard deviation of the per-individual random effect (one standard deviation above the mean) was, therefore, $\sqrt{1.047}=1.023$ (Table 1), indicating that the average spread of the relative risk of leaving was *e*^1.023^ ≈2.78 among individuals. In other words, the per-individual risk of leaving was, on average, 2.20 times higher or lower than the average risk of leaving. However, an inspection of the random effect coefficients per individual showed that the individual effect ranged from 0.1 to more than 6.5 times the average risk but was less than 2 for 44 individuals (77% of the individuals in our study). This means that some individuals were very different in their movements compared to others, which increased the individual variability considerably.

Varying the resampling rate or the cutoff values for separating paths had little effect on the estimated HRs, as well as the predicted risk of leaving (Supplementary 1), suggesting a robust estimation of the risk of leaving.

## 4. Discussion

We designed a new method, based on animal movements, in order to help evaluate how big restricted zones should be to successfully control infectious diseases in wildlife. Our analytical framework is relatively simple to implement and interpret and combines two existing and validated methods implemented in R that do not require restrictive assumptions, extensive parametrizations, or visual interpretation. Coupled with the fact that it solely relies on the movement patterns of the host species, our method can be easily applied to many wildlife-infectious disease systems, if movement data for the target animals are available; for example, to support the establishment of an oral vaccination zone in raccoon rabies management [10, 43] or a control buffer around bovine tuberculosis affected area in possums [8]. A good understanding of the epidemiology of the disease and transmission mechanisms is, however, recommended.

Although the FPT analysis is easily implemented in R (see adehabitatLT package), our approach requires a slight adaption of the function in order to be able to calculate the minimum time spent in a circle when the positions entering and leaving the circle are unknown. Ignoring these times would lead to a loss of information and to an underestimation of the times, and thus an overestimation of the risk of leaving. Using field data of wild boar, we found that modeling FPT-derived data (times spent in circles) with CPH models is robust to low sampling rates (interval up to 4 hr, Supplementary 1); a minimum of six relocations per day might be sufficient to model the risk of leaving restricted zones accordingly, a resolution often achieved by studies monitoring terrestrial mammals to get seasonal and/or multiannual patterns of habitat use. This robustness facilitated the comparison of results between studies, even if location acquisition rates vary. In the wild boar example, the results of the different CPH models were qualitatively similar, which allowed us to use the fixed-effects CPH models for approximate predictions of the individual's risk of leaving an area and to demonstrate the strength of our results. We applied a weighted model to deal with proportional hazard violation and to account for unbalanced sample sizes between individuals [44, 45], but this model ran very slowly (∼24 hr vs. a few minutes for the other two CPH models). Therefore, it may be even less suitable if more data or covariates are included in the model. Finally, the results on wild boar were in agreement with (1) previous findings on the spatial strategies of wild boar [46] and (2) the buffer used between areas of viral circulation and the implementation of barriers during previous ASF epidemics (see below). These results validate the reliability of our approach for determining restricted zones for disease management.

The size and shape of restricted zones are often determined on the basis of (i) the ecology of the host species, such as average annual home range size or distance traveled; or (ii) the expected speed of the epidemic wave [13, 43, 47]. Compared to these methods, our approach has the advantage that it does not require location data over long periods and thus allows the use of datasets that do not incorporate full annual coverage (for example, due to battery failure or loss of the tracking device). Data from a few months, spread over the year if seasonal effects need to be addressed, may be sufficient to estimate the risk of an individual leaving an area and the restricted zones accordingly. This is particularly relevant for hunted species, such as wild boar, for which monitoring may be shortened due to hunting (as in our study). Estimating daily distance traveled requires high-resolution data, which is not the case with our approach. Determining restricted zones based on the expected speed of the epidemic wave demands real epidemic data and a good knowledge of the epidemiology of the infection, which are often not available. Furthermore, the wave velocity will depend on many factors, such as the population density of the host species, the time of virus introduction, the continuity of the suitable habitat for the host species, and the types of management measures deployed [13].

Our approach is based on location data from healthy animals, but this has two advantages: (1) it allows for a conservative estimate of the zone size. Diseased animals generally show reduced movement rates compared to healthy animals, for example, wild boar and domestic pigs infected with ASF [48–50] and swans infected with avian influenza A [51]. Using data from healthy animals may, therefore, overestimate the size of restricted zones required to limit the movement of infected animals, but it increases the chances of containing all infected animals. (2) It makes our method more readily applicable, as collecting location data from infected animals in wild populations is almost impossible. For example, most wild boar infected with the highly virulent strain of ASF die within 7–13 days after infection [23, 24, 52].

Individual movements are an important determinant of disease persistence and spread, and a valuable input for evaluating the effectiveness of various control strategies, such as the width of restricted zones, as shown in our study. How individuals share space and interact with each other is another major factor in explaining the spread of many infectious diseases [53–57] and ultimately affecting restricted zones, a characteristic not taken into account in our method. Restricted zones are also likely to depend on other factors, such as human infrastructures (e.g., settlements, highways, roads) and disturbances (e.g., hunting, recreational activities), the viability and characteristics of the pathogen (e.g., mode of transmission, infectiousness, host diversity), the speed of disease detection compared to the current location of the outbreak, and the type of restrictions and measures (e.g., fences for animal movement restriction) used to control the disease. Continuous surveillance is required to make the appropriate modifications as the epidemiological context changes.

The application of our method to wild boar location data enabled us to make inferences about the size of the buffer to be used to define the infected zone and potential fence imposed during the ASF outbreak. Our results indicate that the risk of leaving an area decreases with increasing radius, but not substantially when the area radius is ≥6 km. Based on this result, a buffer size can be determined that is both effective in controlling the disease and limits conflicts with stakeholders in any future ASF outbreak in our study area. For our wild boar population, we suggest that an initial buffer of 6 km around the outbreak site (∼8,000 ha) might be sufficient to determine the infected zone and contain the disease. As the ASF virus spreads about 0.5–5 km per month depending on wild boar density and human activities [13, 58], the buffer might be increased by 0.5–5 km for each month of delay in detection relative to the current outbreak. A larger initial buffer, e.g., 10 km, would increase the chances of controlling the disease, especially as the probability of virus transmission at such distance is possible but low [56]. However, it would also considerably increase the area where restrictions apply, without much reducing the risk that an infected wild boar had already left the area when the measure was implemented (difference varying from 0.01 to 0.10 over time compared to a distance of 6 km, Supplementary 1). We acknowledge that these results are based on population-level averages and should be treated with caution when applied to management measures. As the individual variability in the risk of leaving an area is large, the few individuals that leave the infected zone (and carry the virus) could make control efforts ineffective. However, infected individuals generally move less than healthy individuals do, thus minimizing this risk. This 6 km distance supports the size of the buffer used in the past to determine the fenced infected zone established to control the ASF epidemic in wild boar in the Czech Republic in 2017 and in Belgium in 2018 [59–63]. In both countries, the ASF epidemic only affected wild boar populations and was successfully controlled [61, 64]. In contrast, a 20 km wide buffer was not sufficient to stop the spread of ASF in Bulgaria, but it should be noted that ASF has been present in both wild boar populations and domestic pig farms [59, 65]. Comparisons of success in ASF control between areas should be made cautiously unless landscape, density, and social and movement behavior of wild boar and measure protocols are similar between areas. In the future, it would be worth developing our approach in an area that is or has been affected by an ASF epidemic to quantitatively assess how the application of this method can help to control the disease.

The risk of leaving an area is dependent upon wild boar sex and season. In general, wild boar had a similar risk of leaving an area of a given size throughout the year, except in spring and winter, when females had a much lower risk of leaving. Previous research has shown seasonal variations in the spatial behavior of female wild boar in the same direction as our results [46]. Spatial and temporal changes in resource availability [35, 66–68] or in hunting pressure [35, 69, 70], as well as reproductive needs [35, 71, 72], could partly explain the observed patterns, but it is beyond the scope of our investigation to provide a comprehensive review of these factors. These results may challenge the current recommendations for controlling wild boar populations and containing the disease of culling mainly females (European Commission [33]), as we found that males are more likely than females to leave an area of a certain size, but only in winter and spring. We acknowledge that the age of individuals likely has a greater influence on the risk of leaving an area [73, 74], but the small sample sizes for the different age-sex classes (in particular, adult males) did not allow this effect to be explored.

Although the landscape in our study area is typical of central Europe, the presented results for wild boar may not be generalizable to any other environment inhabited by wild boar for two main reasons. Our study area is composed largely of croplands, continuous forests, open land, and areas of succession (partly in a National Park), which provide an optimal habitat for wild boar because resources (especially food and resting/sheltering sites) are evenly distributed and readily available. Furthermore, while hunting affects the spatial behavior of wild boar directly by inducing variation in home range size or temporary departures from resting sites [75–77] or indirectly by modifying the population structure through harvesting in favor of a certain age and sex class, in our study area, the hunting pressure is very limited. Both factors may have minimized the movements of wild boar in our study area [78]. The predicted risk of leaving the infected zone in our study population might be lower than in other populations, especially those with a high hunting pressure, such as in France [79]. Nevertheless, our results demonstrate the applicability of the proposed method to any ecosystem where wild boar populations are susceptible to ASF infection and where zonation must be accordingly established. Future work could usefully adapt our method to other wild boar populations under different environmental conditions.

## 5. Conclusions

We outlined a relatively simple and practical method to guide the process of establishing restricted zones of appropriate sizes to contain infectious diseases in wildlife based on the movement data of the host species. Using field data for wild boar, we demonstrated that our new approach is reliable in determining restricted zones, is suitable for large, high-resolution, and irregular location data, and avoids restrictive assumptions, complex parameterizations, and visual interpretation. This work is an example of how to integrate an ecological approach to infection control measures in wildlife, and the method we describe could be broadly applicable for future outbreaks of many diseases. In the future, our approach could be improved by investigating the effect of environmental context on the risk of leaving zones of different sizes [21, 80]. This would help to create or update restricted zones adapted to the environment (e.g., depending on landscape characteristics and host animals densities) rather than using an arbitrary buffer size. In the case of introduction of the ASF virus (our case study) in our study area, our results suggest using a 6 km buffer distance around the area containing all ASF cases to delineate the infected zone and the potential fence. Wherever possible, ASF control by culling should not leave out males, as they have a higher probability of leaving any area in winter and spring and thus spreading the disease.

## Figures and Tables

**Table 1:** Estimated coefficients (*β*) and their standard errors (SE), hazard ratios (e^*β*^), and 95% confidence intervals (CI (e^*β*^)) of the Cox proportional hazard models (with and without random effects) for the covariates.

	Random-effects included			No random-effects included		
Variable	*β* ± SE	HR (e^*β*^)	CI (e^*β*^)	*β* ± SE	HR (e^*β*^)	CI (e^*β*^)
Radius	−0.001 ± NA	0.999	NA	−0.001 ± 0	0.999	0.999–0.999
Sex (female)	0.127 ± NA	1.136	NA	0.037 ± 0.006	1.038	1.026–1.049
Season (spring)	−0.144 ± 0.008	0.866	0.853–0.879	−0.002 ± 0.007	0.998	0.984–1.012
Season (summer)	0.030 ± 0.007	1.030	1.017–1.044	−0.147 ± 0.006	1.158	1.144–1.173
Season (winter)	0.179 ± 0.007	1.197	1.180–1.214	−0.054 ± 0.007	0.947	0.935–0.960
Radius × sex (female)	0 ± 0	1	1.000–1.000	0 ± 0	1	1.000–1.000
Radius × season (spring)	0 ± 0	1	1.000–1.000	0 ± 0	1	1.000–1.000
Radius × season (summer)	0 ± 0	1	1.000–1.000	0 ± 0	1	1.000–1.000
Radius × season (winter)	0 ± 0	1	1.000–1.000	0 ± 0	1	1.000–1.000
Sex (female): season (spring)	−0.533 ± 0.008	0.587	0.578–0.596	−0.566 ± 0.007	0.568	0.560–0.576
Sex (female): season (summer)	−0.191 ± 0.006	0.826	0.816–0.837	−0.149 ± 0.006	0.862	0.852–871
Sex (female): season (winter)	−0.731 ± 0.007	0.481	0.475–0.488	−0.605 ± 0.006	0.546	0.539–0.553
Variance/SD of individual variability	1.047/1.023					

The variance component attributed to individual variability and the standard deviation of the per-individual random effects are also given. Model abbreviations: radius refers to the radius of the circle (in meters); sex is sex of the animal (two categories: male and female); and season is season of the year (four categories: winter, spring, summer and autumn). Male and autumn were considered as reference levels.

## Data Availability

The data that support the findings of this study are available in the EUROBOAR repository (http://euroboar.org), accessible following user registration.

## Supplementary Materials

Supplementary Materials Supplementary 1: The supporting information gives (1) the results of the different approaches to the CPH model, (2) the results of the CPH model for different location data acquisition rates and cutoff values for the separation of individual tracks, and (3) the estimated risk of a wild boar leaving an area at given times and estimated times at fixed risks from the CPH model.

## References

[1] Schlundt J., Kock R. A., Fischer J. R. (2002). Infecious animal diseases: the wildlife/livestock interface. *Revue Scientifique et Technique*.

[2] Bengis R. G., Leighton F. A., Fischer J. R., Artois M., Mörner T., Tate C. M. (2004). The role of wildlife in emerging and re-emerging zoonoses. *Revue Scientifique et Technique*.

[3] Kock R. A., Wambua J. M., Mwandia J. (1999). Rinderpest epidemic in wild ruminants in Kenya 1993–1997. *Veterinary Record*.

[4] Gilchrist M. J., Greko C., Wallinga D. B., Beran G. W., Riley D. G., Thorne P. S. (2007). The potential role of concentrated animal feeding operations in infectious disease epidemics and antibiotic resistance. *Environmental Health Perspectives*.

[5] Vora N. (2008). Impact of anthropogenic environmental alterations on vector-borne diseases. *Medscape Journal of Medicine*.

[6] Carstensen M., DonCarlos M. W. (2011). Preventing the establishment of a wildlife disease reservoir: a case study of bovine tuberculosis in wild deer in Minnesota, USA. *Veterinary Medicine International*.

[7] Jennelle C. S., Walter W. D., Crawford J., Rosenberry C. S., Wallingford B. D. (2022). Movement of white-tailed deer in contrasting landscapes influences management of chronic wasting disease. *The Journal of Wildlife Management*.

[8] Ramsey D. S. L., Efford M. G. (2010). Management of bovine tuberculosis in brushtail possums in New Zealand: predictions from a spatially explicit, individual-based model. *Journal of Applied Ecology*.

[9] Dixon L. K., Stahl K., Jori F., Vial L., Pfeiffer D. U. (2020). African swine fever epidemiology and control. *Annual Review of Animal Biosciences*.

[10] Rosatte R., Donovan D., Allan M. (2001). Emergency response to raccoon rabies introduction into Ontario. *Journal of Wildlife Diseases*.

[11] Mysterud A., Rolandsen C. M. (2019). Fencing for wildlife disease control. *Journal of Applied Ecology*.

[12] Wobeser G. (2002). Disease management strategies for wildlife. *Revue Scientifique et Technique*.

[13] Guberti V., Khomenko S., Masiulis M., Kerba S. (2022). African swine fever in wild boar-ecology and biosecurity. *FAO Animal Production and Health Manual No. 28*.

[14] Kays R., Crofoot M. C., Jetz W., Wikelski M. (2015). Terrestrial animal tracking as an eye on life and planet. *Science*.

[15] Joo R., Boone M. E., Clay T. A., Patrick S. C., Clusella-Trullas S., Basille M. (2020). Navigating through the R packages for movement. *Journal of Animal Ecology*.

[16] Fauchald P., Tveraa T. (2003). Using first-passage time in the analysis of area-restricted search and habitat selection. *Ecology*.

[17] Oksanen S. M., Niemi M., Ahola M. P., Kunnasranta M. (2015). Identifying foraging habitats of Baltic ringed seals using movement data. *Movement Ecology*.

[18] Pinaud D. (2008). Quantifying search effort of moving animals at several spatial scales using first-passage time analysis: effect of the structure of environment and tracking systems. *Journal of Applied Ecology*.

[19] Le Corre M., Dussault C., Côté S. D. (2014). Detecting changes in the annual movements of terrestrial migratory species: using the first-passage time to document the spring migration of caribou. *Movement Ecology*.

[20] Cox D. R. (1972). Regression models and life-tables. *Journal of the Royal Statistical Society, Series B (Methodological)*.

[21] Freitas C., Kovacs K. M., Lydersen C., Ims R. A. (2008). A novel method for quantifying habitat selection and predicting habitat use. *Journal of Applied Ecology*.

[22] Sauter-Louis C., Conraths F. J., Probst C. (2021). African swine fever in wild boar in Europe—a review. *Viruses*.

[23] Blome S., Gabriel C., Dietze K., Breithaupt A., Beer M. (2012). High virulence of African swine fever virus Caucasus isolate in European wild boars of all ages. *Emerging Infectious Diseases*.

[24] Gabriel C., Blome S., Malogolovkin A. (2011). Characterization of African swine fever virus Caucasus isolate in European wild boars. *Emerging Infectious Diseases*.

[25] Gallardo C., Soler A., Nieto R. (2017). Experimental infection of domestic pigs with African swine fever virus Lithuania 2014 genotype II field isolate. *Transboundary and Emerging Diseases*.

[26] Pietschmann J., Guinat C., Beer M. (2015). Course and transmission characteristics of oral low-dose infection of domestic pigs and European wild boar with a Caucasian African swine fever virus isolate. *Archives of Virology*.

[27] Gallardo C., Nurmoja I., Soler A. (2018). Evolution in Europe of African swine fever genotype II viruses from highly to moderately virulent. *Veterinary Microbiology*.

[28] Gallardo C., Soler A., Rodze I. (2019). Attenuated and non-haemadsorbing (non-HAD) genotype II African swine fever virus (ASFV) isolated in Europe, Latvia 2017. *Transboundary and Emerging Diseases*.

[29] Sánchez-Vizcaíno J. M., Mur L., Gomez-Villamandos J. C., Carrasco L. (2015). An update on the epidemiology and pathology of African swine fever. *Journal of Comparative Pathology*.

[30] Zani L., Forth J. H., Forth L. (2018). Deletion at the 5′-end of Estonian ASFV strains associated with an attenuated phenotype. *Scientific Reports*.

[31] Carlson J., Fischer M., Zani L. (2020). Stability of African swine fever virus in soil and options to mitigate the potential transmission risk. *Pathogens*.

[32] Mazur-Panasiuk N., Woźniakowski G. (2020). Natural inactivation of African swine fever virus in tissues: influence of temperature and environmental conditions on virus survival. *Veterinary Microbiology*.

[33] European Commission (EC) (2018). Strategic approach to the management of African swine fever for the EU. SANTE/7113/2015 – rev. Working document. Brussels, European Commission. Directorate general for health and food safety. http://ec.europa.eu/food/sites/food/files/animals/docs/ad_control-measures_asf_wrk-doc-sante-2015-7113.pdf.

[34] Guberti V., Khomenko S., Masiulis M., Kerba S. (2019). African swine fever in wild boar ecology and biosecurity. *FAO Animal Production and Health Manual No. 22*.

[35] Klamm A., Dachs D., Ebert C., Franke U., Henkel A., Morelle K. (2020). Entwicklung und Raumnutzung eines schwarzwild-bestandes in abhängigkeit von den naturräumlichen gegebenheiten des buchenwald-nationalparks hainich und dessen intensiv landwirtschaftlich genutzten umfeldes. Abschlussbericht für das thüringer ministerium für infrastruktur und landwirtschaft. Landesjagdverband thüringen e. V., nationalparkverwaltung hainich und thüringenforst AöR, forstliches forschungs-und kompetenzzentrum.

[36] Brogi R., Brivio F., Bertolucci C. (2019). Capture effects in wild boar: a multifaceted behavioural investigation. *Wildlife Biology*.

[37] Morellet N., Verheyden H., Angibault J.-M., Cargnelutti B., Lourtet B., Hewison M. A. J. (2009). The effect of capture on ranging behaviour and activity of the European roe deer *Capreolus capreolus*. *Wildlife Biology*.

[38] Laguna E., Barasona J. A., Vicente J., Keuling O., Acevedo P. (2021). Differences in wild boar spatial behaviour among land uses and management scenarios in Mediterranean ecosystems. *Science of the Total Environment*.

[39] Therneau T. (2021). A package for survival analysis in R. https://CRAN.R-project.org/package=survival.

[40] Murray D. L. (2006). On improving telemetry-based survival estimation. *The Journal of Wildlife Management*.

[41] Sullivan G. M., Feinn R. (2012). Using effect size-or why the *P* value is not enough. *Journal of Graduate Medical Education*.

[42] R Core Team (2020). *R: A Language and Environment for Statistical Computing*.

[43] Elmore S. A., Chipman R. B., Slate D., Huyvaert K. P., VerCauteren K. C., Gilbert A. T. (2017). Management and modeling approaches for controlling raccoon rabies: the road to elimination. *PLOS Neglected Tropical Diseases*.

[44] Schemper M., Wakounig S., Heinze G. (2009). The estimation of average hazard ratios by weighted Cox regression. *Statistics in Medicine*.

[45] Sitlani C. M., Lumley T., McKnight B. (2020). Incorporating sampling weights into robust estimation of Cox proportional hazards regression model, with illustration in the multi-ethnic study of atherosclerosis. *BMC Medical Research Methodology*.

[46] Thurfjell H., Spong G., Ericsson G. (2014). Effects of weather, season, and daylight on female wild boar movement. *Acta Theriologica*.

[47] Rosatte R. C., Donovan D., Allan M. (2009). The control of raccoon rabies in Ontario Canada: proactive and reactive tactics, 1994–2007. *Journal of Wildlife Diseases*.

[48] Fernández-Carrión E., Barasona J. Á., Sánchez A., Jurado C., Cadenas-Fernández E., Sánchez-Vizcaíno J. M. (2020). Computer vision applied to detect lethargy through animal motion monitoring: a trial on African swine fever in wild boar. *Animals (Basel)*.

[49] Fernández-Carrión E., Martinez-Avilés M., Ivorra B., Martínez-López B., Ramos A. M., Sánchez-Vizcaíno J. M. (2017). Motion-based video monitoring for early detection of livestock diseases: the case of African swine fever. *PLOS ONE*.

[50] Martinez-Avilés M., Fernández-Carrión E., García-Baones J. M. L., Sánchez-Vizcaíno J. M. (2017). Early detection of infection in pigs through an online monitoring system. *Transboundary and Emerging Diseases*.

[51] van Gils J. A., Munster V. J., Radersma R., Liefhebber D., Fouchier R. A., Klaassen M. (2007). Hampered foraging and migratory performance in swans infected with low-pathogenic avian influenza a virus. *PLoS ONE*.

[52] Blome S., Gabriel C., Beer M. (2013). Pathogenesis of African swine fever in domestic pigs and European wild boar. *Virus Research*.

[53] Craft M. E. (2015). Infectious disease transmission and contact networks in wildlife and livestock. *Philosophical Transactions of the Royal Society B: Biological Sciences*.

[54] Pepin K. M., Golnar A., Podgórski T. (2021). Social structure defines spatial transmission of African swine fever in wild boar. *Journal of the Royal Society Interface*.

[55] Podgórski T., Apollonio M., Keuling O. (2018). Contact rates in wild boar populations: implications for disease transmission. *The Journal of Wildlife Management*.

[56] Podgórski T., Pepin K. M., Radko A. (2022). How do genetic relatedness and spatial proximity shape African swine fever infections in wild boar?. *Transboundary and Emerging Diseases*.

[57] Reynolds J. J. H., Hirsch B. T., Gehrt S. D., Craft M. E. (2015). Raccoon contact networks predict seasonal susceptibility to rabies outbreaks and limitations of vaccination. *Journal of Animal Ecology*.

[58] Vaclavek P. (2019). ASF in the Czech Republic: management experience and lessons learnt. *FAO Regional ASF Wild Boar Management Workshop*.

[59] Jori F., Massei G., Licoppe A., Iacolina L., Penrith M.-L., Bellini S. (2021). Management of wild boar populations in the European Union before and during the ASF crisis. *Understanding and Combatting African Swine Fever: A European Perspective*.

[60] EFSA Panel on Animal Health and Welfare (AHAW), More S., Miranda M. A. (2018). African swine fever in wild boar. *EFSA Journal*.

[61] OIE (2019). *Self-Declaration of the Recovery of Freedom from African Swine Fever in All Suids by the Czech Republic*.

[62] Šatrán P. (2019). From ASF infection in wild boar to eradication and free status recovery in the Czech Republic.

[63] Smith G. C., Brough T., Podgórski T. (2022). Defining and testing a wildlife intervention framework for exotic disease control. *Ecological Solutions and Evidence*.

[64] OIE (2020). *Self-Declaration of Belgium’s African Swine Fever-Free Status in All Swine Species*.

[65] Zani L., Dietze K., Dimova Z. (2019). African swine fever in a Bulgarian backyard farm—a case report. *Veterinary Sciences*.

[66] Geisser H., Reyer H.-U. (2005). The influence of food and temperature on population density of wild boar *Sus scrofa* in the Thurgau (Switzerland). *Journal of Zoology*.

[67] Keuling O., Stier N., Roth M. (2009). Commuting, shifting or remaining? Different spatial utilisation patterns of wild boar *Sus scrofa* L. in forest and field crops during summer. *Mammalian Biology*.

[68] Schley L., Roper T. J. (2003). Diet of wild boar *Sus scrofa* in Western Europe, with particular reference to consumption of agricultural crops. *Mammal Review*.

[69] Rosell C., Navàs F., Romero S., de Dalmases I. (2004). Activity patterns and social organization of wild boar (*Sus scrofa* L.) in a wetland envrionment: preliminary data on the effects of shooting individuals. *Galemys*.

[70] Tolon V., Dray S., Loison A., Zeileis A., Fischer C., Baubet E. (2009). Responding to spatial and temporal variations in predation risk: space use of a game species in a changing landscape of fear. *Canadian Journal of Zoology*.

[71] Gamelon M., Focardi S., Baubet E. (2017). Reproductive allocation in pulsed-resource environments: a comparative study in two populations of wild boar. *Oecologia*.

[72] Gethöffer F., Sodeikat G., Pohlmeyer K. (2007). Reproductive parameters of wild boar (*Sus scrofa*) in three different parts of Germany. *European Journal of Wildlife Research*.

[73] Keuling O., Lauterbach K., Stier N., Roth M. (2010). Hunter feedback of individually marked wild boar *Sus scrofa* L.: dispersal and efficiency of hunting in northeastern Germany. *European Journal of Wildlife Research*.

[74] Keuling O., Stier N., Roth M. (2008). Annual and seasonal space use of different age classes of female wild boar *Sus scrofa* L. *European Journal of Wildlife Research*.

[75] Calenge C., Maillard D., Vassant J., Brandt S. (2002). Summer and hunting season home ranges of wild boar (*Sus scrofa*) in two habitats in France. *Game and Wildlife Science*.

[76] Sodeikat G., Pohlmeyer K. (2002). Temporary home range modifications of wild boar family groups (*Sus scrofa* L.) caused by drive hunts in lower saxony (Germany). *Zeitschrift für Jagdwissenschaft*.

[77] Sodeikat G., Pohlmeyer K. (2007). Impact of drive hunts on daytime resting site areas of wild boar family groups (*Sus scrofa* L.). *Wildlife Biology in Practice*.

[78] Podgórski T., Baś G., Jędrzejewska B. (2013). Spatiotemporal behavioral plasticity of wild boar (*Sus scrofa*) under contrasting conditions of human pressure: primeval forest and metropolitan area. *Journal of Mammalogy*.

[79] Servanty S., Gaillard J.-M., Toïgo C., Brandt S., Baubet E. (2009). Pulsed resources and climate-induced variation in the reproductive traits of wild boar under high hunting pressure. *Journal of Animal Ecology*.

[80] Byrne M. E., Guthrie J. D., Hardin J., Collier B. A., Chamberlain M. J. (2014). Evaluating wild Turkey movement ecology: an example using first-passage time analysis. *Wildlife Society Bulletin*.

