# Supplementary material for: First-Passage Time Analysis Based on GPS Data Offers a New Approach to Estimate Restricted Zones for the Management of Infectious Diseases in Wildlife: A Case Study Using the Example of African Swine Fever
Source: Transbound Emerg Dis. 2023 Aug 25;2023:4024083. doi: 10.1155/2023/4024083 (PMC12017023; doi:10.1155/2023/4024083)
Supplement: Supplementary Materials — Supplementary 1: The supporting information gives (1) the results of the different approaches to the CPH model, (2) the results of the CPH model for different location data acquisition rates and cutoff values for the separation of individual tracks, and (3) the estimated risk of a wild boar leaving an area at given times and estimated times at fixed risks from the CPH model. [file 4024083.f1.docx]

**SUPPORTING INFORMATION**

1. *Comparison of Cox proportional hazard models fitted to first-passage time data for the movement paths of 57 wild boar in Hainich National Park, Germany, 2017–2019.*

Our approach implicitly involves repeated observations for individuals as the time spent in a circle is calculated for each radius size and each location of an animal’s trajectory. If the inherent variability at the individual level is not considered, the data of animals with a greater number of locations, and therefore values of time spent in a circle, could bias the results. However, the development of mixed-effects CPH models is very recent, and, in many respects, still at an early stage, such that some functions have yet to be implemented in the available software (Table S1).

We present and compare the different CPH models developed to account for repeated and non-independent observations from the same individual. We built a mixed-effect CPH model with individual identity as a random factor, using the ‘coxme’ package in R. As survival function S(*t*) that use random-effects CPH models to predict the probability of an animal staying in an area longer than a time *t* have not yet been implemented in R, we fitted a fixed-effects CPH model. We checked the proportional hazards assumption, required by CPH models, using the fixed-effects CPH model, as it is not yet implemented for the mixed-effects CPH models (Table S1). Verification was performed by checking the scaled Schoenfeld residuals, both visually and by testing whether their slope was zero (not shown). None of the variables used in the model satisfied this assumption. To reduce the impact of violating the model assumption and to account for repeated observations and unbalanced sample sizes between individuals (Schemper et al., 2009; Sitlani et al., 2020), we also built a weighted CPH model, in which the weights of individuals with *k* events were reduced by giving them a weight of 1/*k* for each data point. We compared hazard ratios (HRs) using the exponential of each coefficient between the different models.

While the proportional hazard assumption was not verified in the fixed-effects CPH model, all variables used in the weighted CPH model satisfied this assumption (not shown). The fixed-effects CPH model therefore estimates average hazard over time, whereas the weighted model gives proportional HRs over time. Despite of this discrepancy, there was, in general, good consistency between β coefficients of the different CPH models (Table S2). The effect of radius size on the risk of leaving is the same regardless of the model built. The largest difference between model types resulted from the effect of season, in which case the HR was generally lower in the mixed-effect than in the fixed-effect or equally-weighted CPH models, but the direction of the effect was similar (all HR > 1 or all HR < 1, Table S2, Figure S1). Although the weighted CPH model seems the most convenient (model assumption verified and unbalanced sample sizes considered), it was slow to run (about 24 hours vs. a few minutes for the fixed- and mixed-effects CPH models), which may make it unpractical in the future if more data or covariates are included in the model. For this reason and because coefficients are very similar between the models, we based our interpretations mainly on the mixed-effects CPH model, but when necessary (such as to predict the risk of leaving an area over time, Table S1), we used the fixed-effects CPH model in the main text. It is worth pointing out that violation of the non-proportionality assumption alone does not automatically lead to biased estimates and non-proportionality is not an issue (Kuitunen et al., 2021).

**Table S1.** The functions available for each type of model.

|  | Repeated observations | CPH assumption | Survival curves |
| --- | --- | --- | --- |
| No-random effects | No | Yes | Yes |
| Random effects | Yes | No | No |
| Equally weighted individuals | Yes | Yes | Yes |


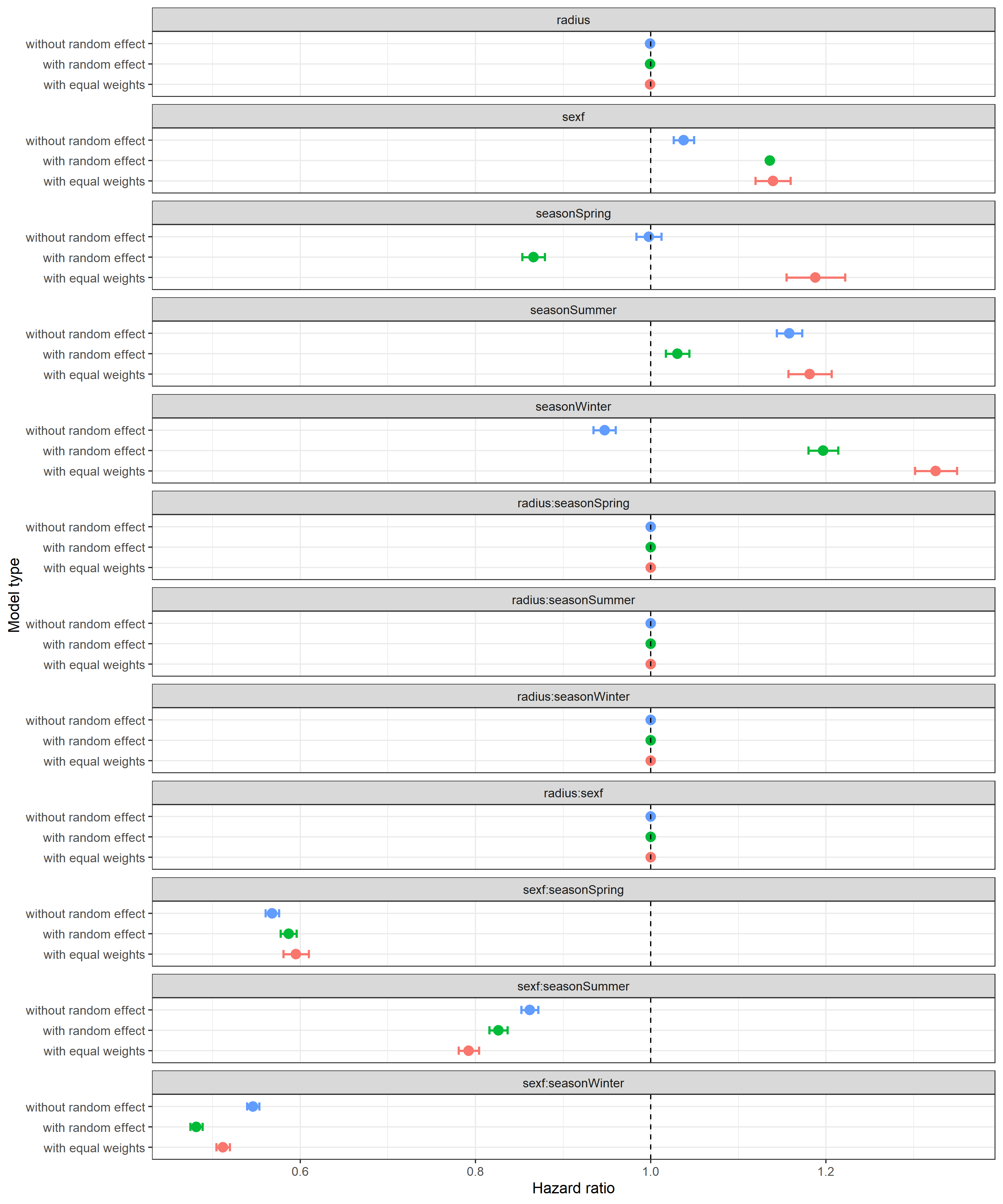


**Figure S1**. Estimated hazard ratios (e^β^) and 95% confidence intervals of the different CPH models (with or without random effects, or with the same weights for all individuals) for the covariates.

**Table S2.** Estimated coefficients (β), hazard ratios (e^β^), and 95% confidence intervals [CI (e^β^)] of the CPH models for the covariates. The different models were constructed with and without random effects for individual identity, and with the same weight for all individuals.

|  | Equally-weighted individuals | | | Random-effects included | | | No random effects included | | |
| --- | --- | --- | --- | --- | --- | --- | --- | --- | --- |
| Variable | β | HR (e^β^) | CI (e^β^) | β | HR (e^β^) | CI (e^β^) | β | HR (e^β^) | CI (e^β^) |
| Radius | -0.001 | 0.999 | 0.999-0.999 | -0.001 | 0.999 | NA | -0.001 | 0.999 | 0.999-0.999 |
| Sex [Female] | 0.130 | 1.139 | 1.119-1.159 | 0.127 | 1.136 | NA | 0.037 | 1.038 | 1.026-1.049 |
| Season [Spring] | 0.172 | 1.188 | 1.155-1.222 | -0.144 | 0.866 | 0.853-0.879 | -0.002 | 0.998 | 0.984-1.012 |
| Season [Summer] | 0.167 | 1.181 | 1.157-1.206 | 0.030 | 1.030 | 1.017-1.044 | -0.147 | 1.158 | 1.144-1.173 |
| Season [Winter] | 0.282 | 1.325 | 1.301-1.349 | 0.179 | 1.197 | 1.180-1.214 | -0.054 | 0.947 | 0.935-0.960 |
| Radius x sex [Female] | 0.000 | 1.000 | 1.000-1.000 | 0.000 | 1.000 | 1.000-1.000 | 0.000 | 1.000 | 1.000-1.000 |
| Radius x season [Spring] | 0.000 | 1.000 | 1.000-1.000 | 0.000 | 1.000 | 1.000-1.000 | 0.000 | 1.000 | 1.000-1.000 |
| Radius x season [Summer] | 0.000 | 1.000 | 1.000-1.000 | 0.000 | 1.000 | 1.000-1.000 | 0.000 | 1.000 | 1.000-1.000 |
| Radius x season [Winter] | 0.000 | 1.000 | 1.000-1.000 | 0.000 | 1.000 | 1.000-1.000 | 0.000 | 1.000 | 1.000-1.000 |
| Sex [Female]: season [Spring] | -0.159 | 0.595 | 0.581-0.610 | -0.533 | 0.587 | 0.578-0.596 | -0.566 | 0.568 | 0.560-0.576 |
| Sex [Female]: season [Summer] | -0.233 | 0.792 | 0.781-0.804 | -0.191 | 0.826 | 0.816-0.837 | -0.149 | 0.862 | 0.852-871 |
| Sex [Female]: season [Winter] | -0.670 | 0.512 | 0.504-0.520 | -0.731 | 0.481 | 0.475-0.488 | -0.605 | 0.546 | 0.539-0.553 |

Model abbreviations: radius refers to the radius of the circle (in meters); sex is sex of the animal (2 categories: male and female); and season is season of the year (4 categories: winter, spring, summer and autumn). Male and autumn were considered as reference levels.

1. *Comparison of CPH model results for varying acquisition rates of location data or different cut-off values for splitting individual tracks*

How often GPS fixes are acquired can definitely influence the times spent in the circles calculated with the FPT analysis, and therefore the predicted risk of leaving. We evaluate the generality of our approach for different temporal scales by comparing the results of CPH models using datasets with locations collected at different time intervals. To do that, we down-sampled the original data from 30-min to 1-, 2-, 3-, and 4-hour and repeated our approach on each resampled dataset (FPT analysis and CPH models, see main text for further explanation). These time intervals were chosen because they are the most common resolution for fine-scale studies. Similarly, we evaluate how robust our estimates of the hazard ratios and the predicted risk of leaving were to changes in the time values used to define the number of acceptable missing locations in the individual’s track. To do this, we used the original 30-minute data and removed any gaps in each individual’s location data by splitting the track into several segments when the time between two successive locations was > 1, 2, 3 (our final analysis), 4, 5 and 6 hours. We then repeated our approach on each new dataset (FPT analysis and CPH models).


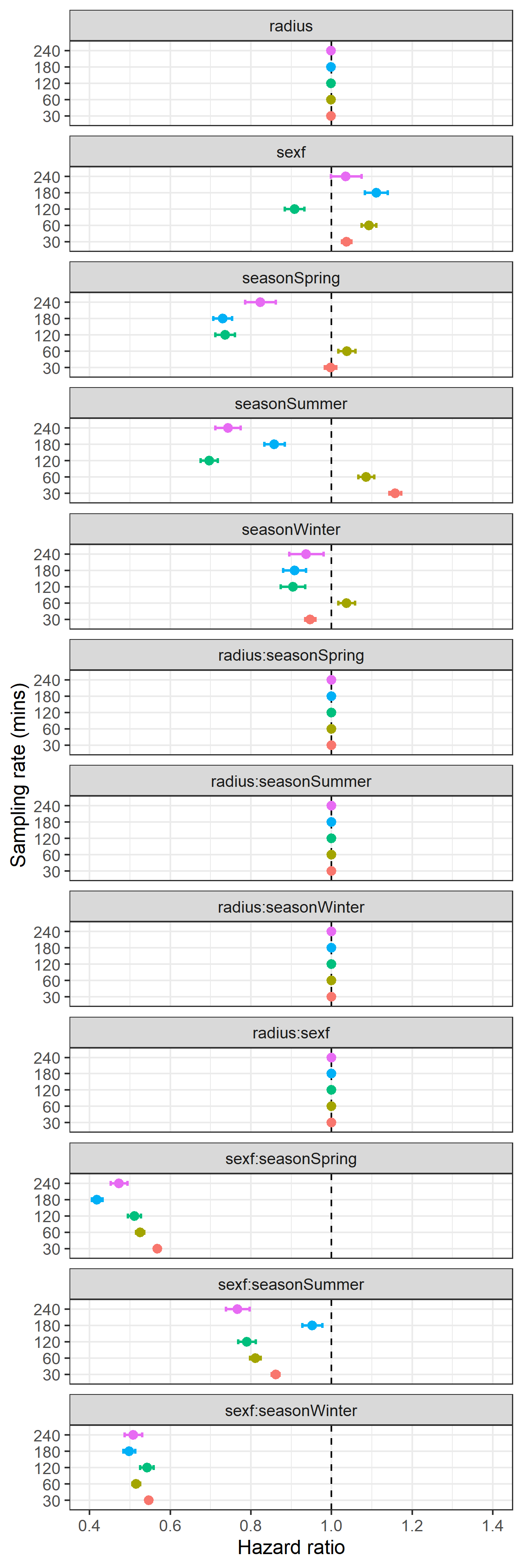


(b)


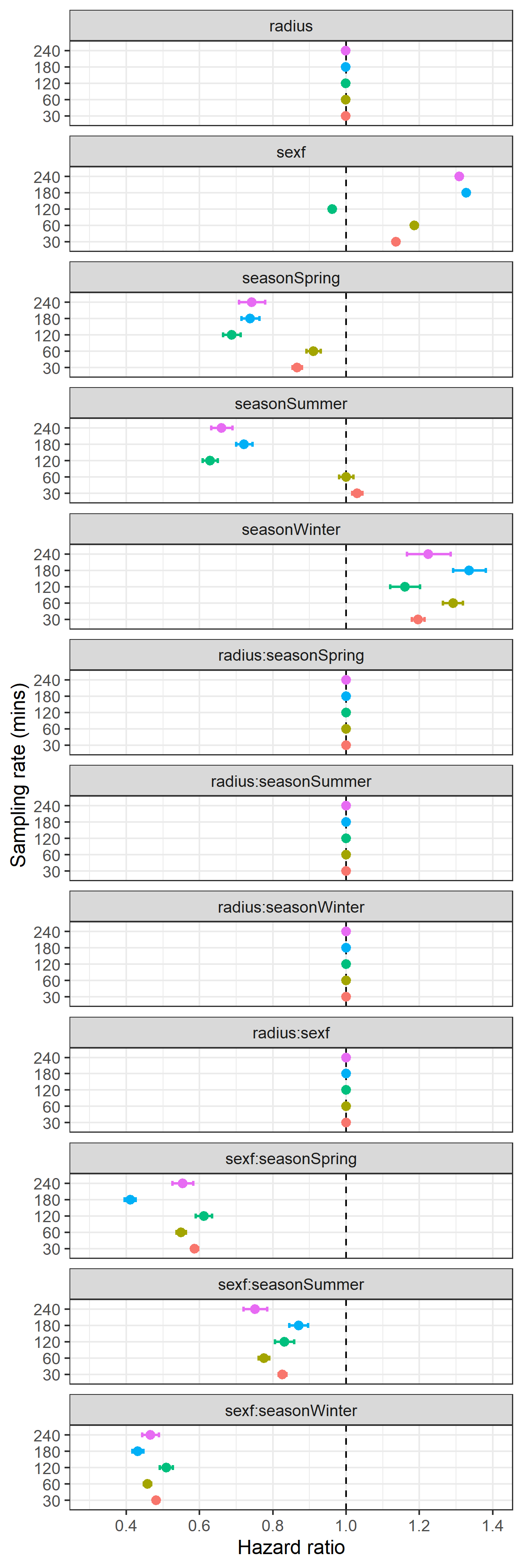


(a)

**Figure S2**. The effect of varying the resampling rate on the hazard ratios (e^β^) for the different covariates estimated from (a) the standard fixed-effects CPH model and (b) the mixed-effects CPH model. Horizontal bars indicate 95% confidence intervals.


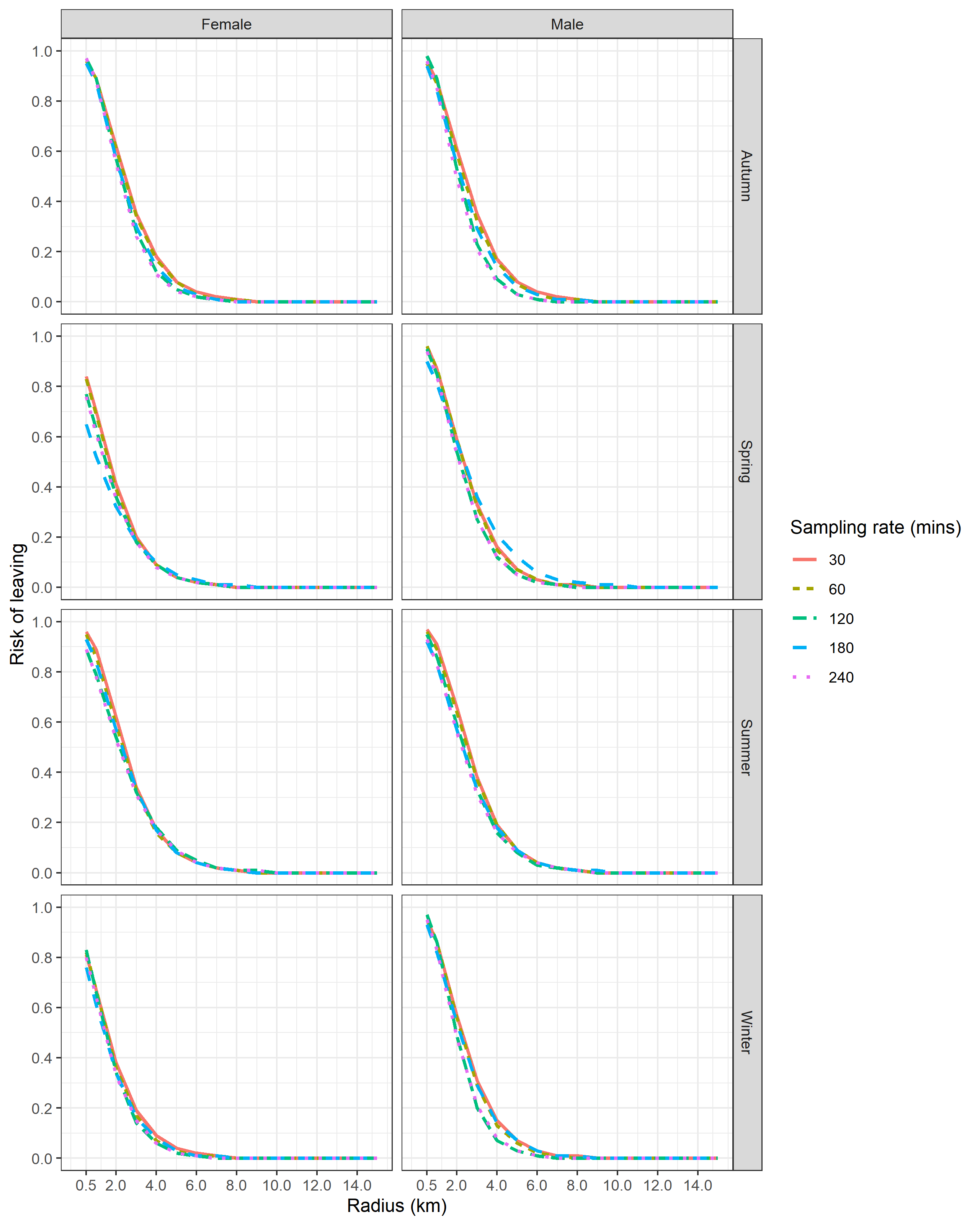


**Figure S3.** The effect of varying the resampling rate on the predicted risk of leaving at *t* = 14 days depending on the radius size, season, and sex of the animal. The predictions were based on the fixed-effects CPH model. We predicted the risk of leaving at *t* = 14 days because most wild boar infected with the highly virulent strain of ASF die within 2 weeks post-infection. The risk of leaving does not differ much according to the sampling rate.

**Figure S4**. The effect of varying the cut-off values for separating paths on the hazard ratios (e^β^) for the different covariates estimated from (a) the standard fixed-effects CPH model and (b) the mixed-effects CPH model. Horizontal bars indicate 95% confidence intervals.


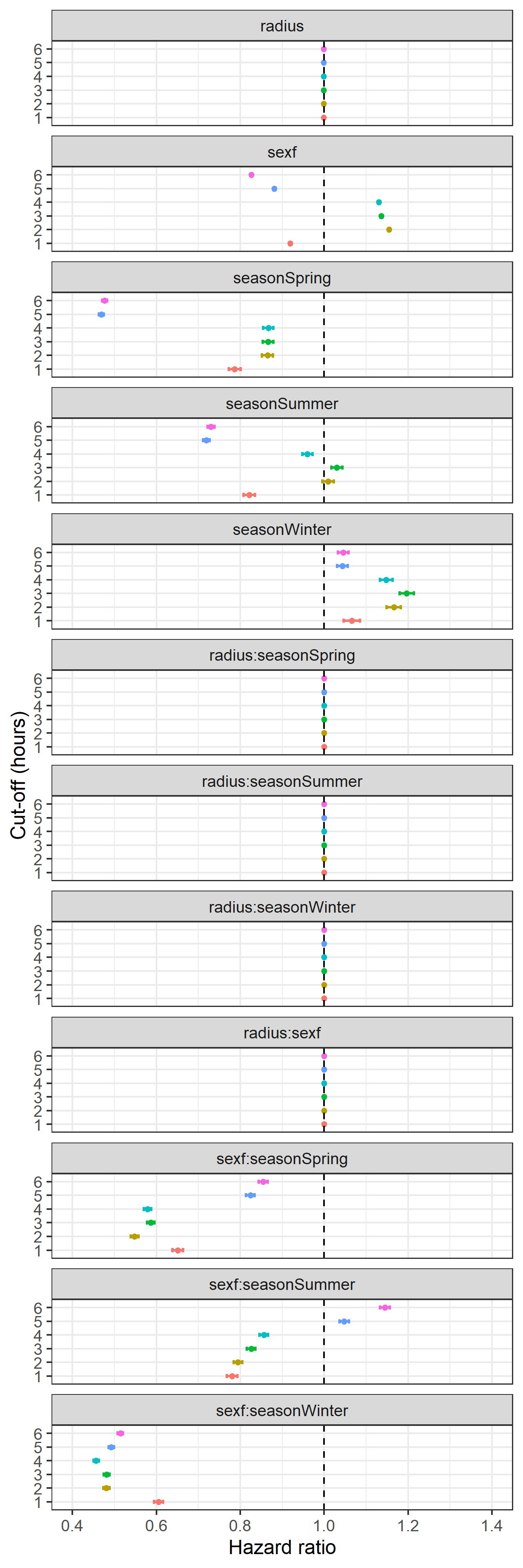


(b)


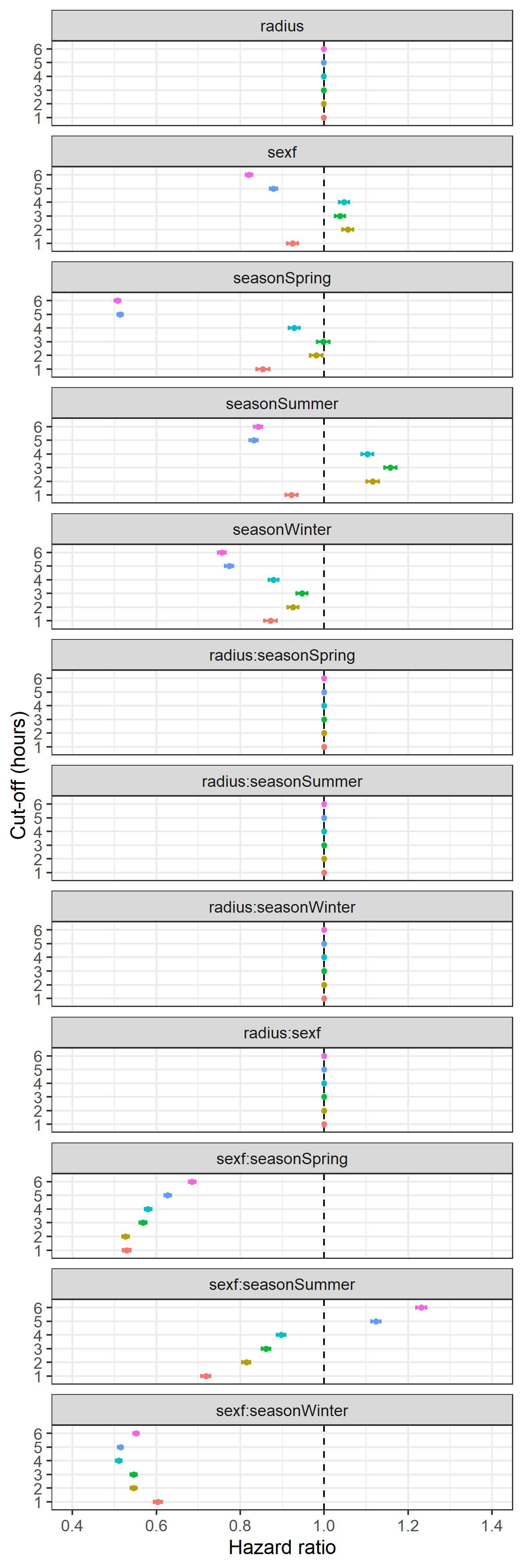


(a)


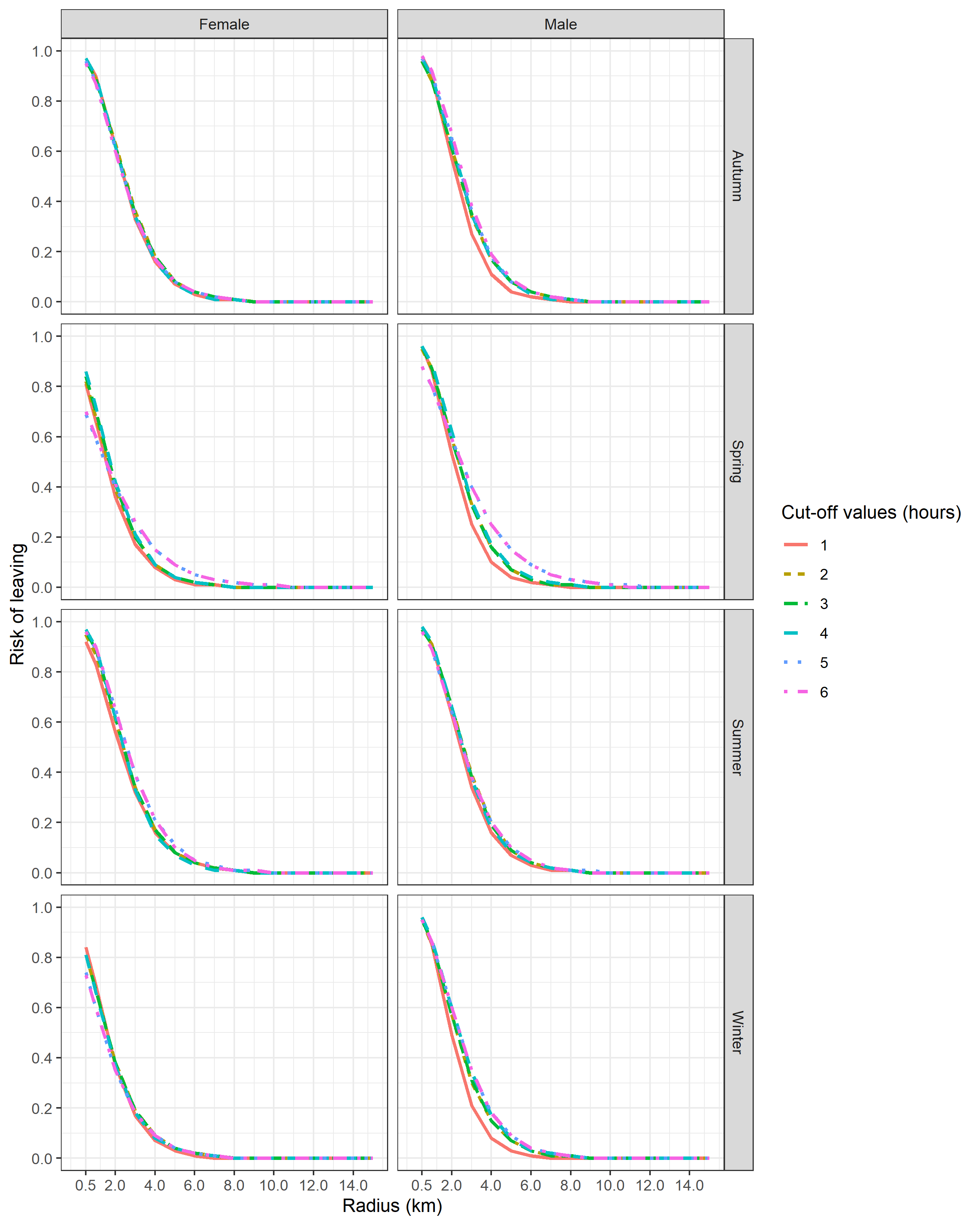


**Figure S5.** The effect of varying the cut-off values for separating paths on the predicted risk of leaving at *t* = 14 days depending on the radius size, season, and sex of the animal. The predictions were based on the fixed-effects CPH model. We predicted the risk of leaving at *t* = 14 days because most wild boar infected with the highly virulent strain of ASF die within 2 weeks post-infection. The risk of leaving does not differ much according to the cut-off values.

1. *Estimated risk of a wild boar leaving an area at given times and estimated times at fixed risks from the CPH model*

As information for disease managers, we calculated the values of the risks of a wild boar leaving an area at given times, and times at fixed risks. These values were estimated from the fixed-effects CPH model (see main text for more details), as the calculation of these functions from the mixed-effects CPH model is not yet implemented (Table S1) and can be used to guide the process of establishing an ASF infection zone of appropriate size.

If an individual is found dead due to ASF, we can estimate the probability that the individual travelled a certain distance during the period of infection, and thus assess the extent of the area potentially contaminated by the individual. For example, assuming that an individual found dead carried the virus for 15 days before dying, the risk that the individual left the 1-km zone surrounding the point of death is ~0.7-0.9 depending on the season (Table S3). The risk decreases to 0.20-0.35 for a 3-km radius surrounding area and to 0.02-0.04 for a 6-km radius area (Table S3).

If a carcass is found that is x days old, one can also determine the likelihood that individuals in **direct contact** with the carcass before it was removed have remained within the surrounding x-kms, or conversely, have left. For example, assuming that an infected carcass has remained in the environment for 10 days, the risk that individuals in direct contact with the carcass at least once during these 10 days have travelled more than 3 km from the carcass site (a radius of 3 km drawn around the carcass) is ≤ 0.15-0.30 depending on the season (Table S3). An individual having been in direct contact with the carcass on day 1 has a risk of having left the surrounding 3 km area of 0.30 after 10 days, but an individual having been in contact with the carcass on day 9 will have a risk of leaving the surrounding area of 0.08 on day 10 of carcass persistence, i.e. the risk after 1 day (Table S3). In the same way, the risk that individuals in direct contact at least once with an infected carcass will travel a distance ≥ 6 km from the location of the carcass (the distance used in past epidemics in the Czech Republic and Belgium for the buffer width) during the 10 days of carcass persistence is ≤ 0. 03; again, an individual having been in contact on day 9 will have a risk of having left the surrounding 6-km zone of only 0.01 on day 10 (risk after 1 day, Table S3).

**Table S3.** Estimated risk of a wild boar leaving an area with a radius of 1 to 20 km after 1 day, 5 days, 10 days, 15 days, 20 days, and 30 days. Predictions are estimated by season, and whether the animal is a female or a male. Predictions are based on the results of the fixed-effects CPH model. The risk varies between 0 (no risk that the animal will leave the given area at the given time) and 1 (the animal will leave the area).

|  | Risk of leaving | | | | | | | | | | | | |
| --- | --- | --- | --- | --- | --- | --- | --- | --- | --- | --- | --- | --- | --- |
|  | Female | | | | | |  | Male | | | | | |
| Radius (km) | 1 day | 5  days | 10 days | 15 days | 20 days | 30 days |  | 1 day | 5  days | 10 days | 15 days | 20 days | 30 days |
| *Summer* |  |  |  |  |  |  |  |  |  |  |  |  |  |
| 1 | 0.37 | 0.74 | 0.84 | 0.90 | 0.93 | 0.95 |  | 0.40 | 0.78 | 0.87 | 0.92 | 0.95 | 0.97 |
| 2 | 0.18 | 0.44 | 0.55 | 0.63 | 0.68 | 0.73 |  | 0.20 | 0.49 | 0.60 | 0.67 | 0.73 | 0.78 |
| 3 | 0.08 | 0.23 | 0.30 | 0.35 | 0.40 | 0.44 |  | 0.10 | 0.25 | 0.33 | 0.39 | 0.44 | 0.48 |
| 4 | 0.04 | 0.11 | 0.14 | 0.17 | 0.20 | 0.23 |  | 0.04 | 0.12 | 0.16 | 0.20 | 0.22 | 0.25 |
| 5 | 0.02 | 0.05 | 0.07 | 0.08 | 0.09 | 0.11 |  | 0.02 | 0.06 | 0.08 | 0.09 | 0.11 | 0.12 |
| 6 | 0.01 | 0.02 | 0.03 | 0.04 | 0.04 | 0.05 |  | 0.01 | 0.03 | 0.03 | 0.04 | 0.05 | 0.06 |
| 7 | 0.00 | 0.01 | 0.01 | 0.02 | 0.02 | 0.02 |  | 0.00 | 0.01 | 0.02 | 0.02 | 0.02 | 0.02 |
| 8 | 0.00 | 0.00 | 0.01 | 0.01 | 0.01 | 0.01 |  | 0.00 | 0.00 | 0.01 | 0.01 | 0.01 | 0.01 |
| 9 | 0.00 | 0.00 | 0.00 | 0.00 | 0.00 | 0.00 |  | 0.00 | 0.00 | 0.00 | 0.00 | 0.00 | 0.00 |
| 10 | 0.00 | 0.00 | 0.00 | 0.00 | 0.00 | 0.00 |  | 0.00 | 0.00 | 0.00 | 0.00 | 0.00 | 0.00 |
| 15 | 0.00 | 0.00 | 0.00 | 0.00 | 0.00 | 0.00 |  | 0.00 | 0.00 | 0.00 | 0.00 | 0.00 | 0.00 |
| 20 | 0.00 | 0.00 | 0.00 | 0.00 | 0.00 | 0.00 |  | 0.00 | 0.00 | 0.00 | 0.00 | 0.00 | 0.00 |
| *Autumn* |  |  |  |  |  |  |  |  |  |  |  |  |  |
| 1 | 0.37 | 0.74 | 0.84 | 0.90 | 0.93 | 0.95 |  | 0.36 | 0.73 | 0.84 | 0.89 | 0.92 | 0.95 |
| 2 | 0.19 | 0.45 | 0.56 | 0.64 | 0.69 | 0.74 |  | 0.18 | 0.44 | 0.55 | 0.63 | 0.68 | 0.73 |
| 3 | 0.09 | 0.23 | 0.31 | 0.36 | 0.41 | 0.45 |  | 0.09 | 0.23 | 0.30 | 0.36 | 0.40 | 0.44 |
| 4 | 0.04 | 0.11 | 0.15 | 0.18 | 0.21 | 0.23 |  | 0.04 | 0.11 | 0.15 | 0.18 | 0.20 | 0.23 |
| 5 | 0.02 | 0.05 | 0.07 | 0.08 | 0.10 | 0.11 |  | 0.02 | 0.05 | 0.07 | 0.08 | 0.10 | 0.11 |
| 6 | 0.01 | 0.02 | 0.03 | 0.04 | 0.04 | 0.05 |  | 0.01 | 0.02 | 0.03 | 0.04 | 0.04 | 0.05 |
| 7 | 0.00 | 0.01 | 0.01 | 0.02 | 0.02 | 0.02 |  | 0.00 | 0.01 | 0.01 | 0.02 | 0.02 | 0.02 |
| 8 | 0.00 | 0.00 | 0.01 | 0.01 | 0.01 | 0.01 |  | 0.00 | 0.00 | 0.01 | 0.01 | 0.01 | 0.01 |
| 9 | 0.00 | 0.00 | 0.00 | 0.00 | 0.00 | 0.00 |  | 0.00 | 0.00 | 0.00 | 0.00 | 0.00 | 0.00 |
| 10 | 0.00 | 0.00 | 0.00 | 0.00 | 0.00 | 0.00 |  | 0.00 | 0.00 | 0.00 | 0.00 | 0.00 | 0.00 |
| 15 | 0.00 | 0.00 | 0.00 | 0.00 | 0.00 | 0.00 |  | 0.00 | 0.00 | 0.00 | 0.00 | 0.00 | 0.00 |
| 20 | 0.00 | 0.00 | 0.00 | 0.00 | 0.00 | 0.00 |  | 0.00 | 0.00 | 0.00 | 0.00 | 0.00 | 0.00 |
| *Winter* |  |  |  |  |  |  |  |  |  |  |  |  |  |
| 1 | 0.21 | 0.49 | 0.61 | 0.68 | 0.74 | 0.78 |  | 0.34 | 0.70 | 0.81 | 0.87 | 0.91 | 0.93 |
| 2 | 0.10 | 0.26 | 0.33 | 0.39 | 0.44 | 0.48 |  | 0.16 | 0.41 | 0.51 | 0.59 | 0.64 | 0.69 |
| 3 | 0.04 | 0.12 | 0.16 | 0.19 | 0.22 | 0.25 |  | 0.08 | 0.20 | 0.27 | 0.32 | 0.36 | 0.40 |
| 4 | 0.02 | 0.05 | 0.07 | 0.09 | 0.10 | 0.12 |  | 0.03 | 0.09 | 0.13 | 0.15 | 0.18 | 0.20 |
| 5 | 0.01 | 0.02 | 0.03 | 0.04 | 0.05 | 0.05 |  | 0.01 | 0.04 | 0.06 | 0.07 | 0.08 | 0.09 |
| 6 | 0.00 | 0.01 | 0.01 | 0.02 | 0.02 | 0.02 |  | 0.01 | 0.02 | 0.03 | 0.03 | 0.04 | 0.04 |
| 7 | 0.00 | 0.00 | 0.01 | 0.01 | 0.01 | 0.01 |  | 0.00 | 0.01 | 0.01 | 0.01 | 0.02 | 0.02 |
| 8 | 0.00 | 0.00 | 0.00 | 0.00 | 0.00 | 0.00 |  | 0.00 | 0.00 | 0.00 | 0.01 | 0.01 | 0.01 |
| 9 | 0.00 | 0.00 | 0.00 | 0.00 | 0.00 | 0.00 |  | 0.00 | 0.00 | 0.00 | 0.00 | 0.00 | 0.00 |
| 10 | 0.00 | 0.00 | 0.00 | 0.00 | 0.00 | 0.00 |  | 0.00 | 0.00 | 0.00 | 0.00 | 0.00 | 0.00 |
| 15 | 0.00 | 0.00 | 0.00 | 0.00 | 0.00 | 0.00 |  | 0.00 | 0.00 | 0.00 | 0.00 | 0.00 | 0.00 |
| 20 | 0.00 | 0.00 | 0.00 | 0.00 | 0.00 | 0.00 |  | 0.00 | 0.00 | 0.00 | 0.00 | 0.00 | 0.00 |
| *Spring* |  |  |  |  |  |  |  |  |  |  |  |  |  |
| 1 | 0.23 | 0.53 | 0.64 | 0.72 | 0.77 | 0.81 |  | 0.35 | 0.72 | 0.83 | 0.88 | 0.92 | 0.94 |
| 2 | 0.11 | 0.28 | 0.36 | 0.42 | 0.47 | 0.52 |  | 0.17 | 0.43 | 0.53 | 0.61 | 0.66 | 0.71 |
| 3 | 0.05 | 0.13 | 0.18 | 0.21 | 0.24 | 0.27 |  | 0.08 | 0.21 | 0.28 | 0.33 | 0.38 | 0.42 |
| 4 | 0.02 | 0.06 | 0.08 | 0.10 | 0.11 | 0.13 |  | 0.04 | 0.10 | 0.13 | 0.16 | 0.19 | 0.21 |
| 5 | 0.01 | 0.03 | 0.04 | 0.04 | 0.05 | 0.06 |  | 0.02 | 0.04 | 0.06 | 0.07 | 0.09 | 0.10 |
| 6 | 0.00 | 0.01 | 0.02 | 0.02 | 0.02 | 0.03 |  | 0.01 | 0.02 | 0.03 | 0.03 | 0.04 | 0.04 |
| 7 | 0.00 | 0.00 | 0.01 | 0.01 | 0.01 | 0.01 |  | 0.00 | 0.01 | 0.01 | 0.01 | 0.02 | 0.02 |
| 8 | 0.00 | 0.00 | 0.00 | 0.00 | 0.00 | 0.00 |  | 0.00 | 0.00 | 0.01 | 0.01 | 0.01 | 0.01 |
| 9 | 0.00 | 0.00 | 0.00 | 0.00 | 0.00 | 0.00 |  | 0.00 | 0.00 | 0.00 | 0.00 | 0.00 | 0.00 |
| 10 | 0.00 | 0.00 | 0.00 | 0.00 | 0.00 | 0.00 |  | 0.00 | 0.00 | 0.00 | 0.00 | 0.00 | 0.00 |
| 15 | 0.00 | 0.00 | 0.00 | 0.00 | 0.00 | 0.00 |  | 0.00 | 0.00 | 0.00 | 0.00 | 0.00 | 0.00 |
| 20 | 0.00 | 0.00 | 0.00 | 0.00 | 0.00 | 0.00 |  | 0.00 | 0.00 | 0.00 | 0.00 | 0.00 | 0.00 |

**Table S4.** Timespan (in days) with 95% confidence intervals (CI) when the estimated risk of a wild boar leaving an area with a radius of 1 to 20 km reaches 0.05 and 0.10. Predictions are estimated by season, and whether the animal is a female or a male. Predictions are based on the results of the fixed-effects CPH model. *na*, means that the risk in question (0.05 or 0.10) was never reached during the entire observation period.

|  | Female | | | | |  | Male | | | | |
| --- | --- | --- | --- | --- | --- | --- | --- | --- | --- | --- | --- |
|  | risk of leaving = 0.05 | |  | risk of leaving = 0.10 | |  | risk of leaving = 0.05 | |  | risk of leaving = 0.10 | |
| Radius (km) | Timespan [CI] (in days) | |  | Timespan [CI] (in days) | |  | Timespan [CI] (in days) | |  | Timespan [CI] (in days) | |
| *Summer* |  |  |  |  |  |  | 0.2 | [0.2-0.2] |  | 0.4 | [0.4-0.4] |
| 1 | 0.3 | [0.3-0.3] |  | 0.5 | [0.5-0.5] |  | 0.5 | [0.5-0.5] |  | 0.8 | [0.8-0.8] |
| 2 | 0.6 | [0.6-0.6] |  | 0.8 | [0.8-0.8] |  | 0.8 | [0.8-0.8] |  | 1.0 | [1.0-1.0] |
| 3 | 0.8 | [0.8-0.8] |  | 1.3 | [1.3-1.3] |  | 1.2 | [1.2-1.2] |  | 3.5 | [3.4-3.6] |
| 4 | 1.7 | [1.7-1.7] |  | 4.6 | [4.4-4.7] |  | 4.1 | [4.0-4.1] |  | 17.5 | [17.0-17.9] |
| 5 | 5.3 | [5.1-5.4] |  | 24.1 | [23.8-25.0] |  | 22.2 | [22.0-23.0] |  | 115.0 | [112.8-115.0] |
| 6 | 31.9 | [31.9-31.9] |  | 147.6 | [145.1-147.9] |  | 145.0 | [145.0-145.0] |  | *na* | *na* |
| 7 | 156.1 | [156.1-156.1] |  | *na* | *na* |  | *na* | *na* |  | *na* | *na* |
| 8 | *na* | *na* |  | *na* | *na* |  | *na* | *na* |  | *na* | *na* |
| 9 | *na* | *na* |  | *na* | *na* |  | *na* | *na* |  | *na* | *na* |
| 10 | *na* | *na* |  | *na* | *na* |  | *na* | *na* |  | *na* | *na* |
| 15 | *na* | *na* |  | *na* | *na* |  | *na* | *na* |  | *na* | *na* |
| 20 | *na* | *na* |  | *na* | *na* |  | *na* | *na* |  | *na* | *na* |
| *Autumn* |  |  |  |  |  |  |  |  |  |  |  |
| 1 | 0.3 | [0.3-0.3] |  | 0.5 | [0.5-0.5] |  | 0.3 | [0.3-0.3] |  | 0.5 | [0.5-0.5] |
| 2 | 0.6 | [0.6-0.6] |  | 0.8 | [0.8-0.8] |  | 0.6 | [0.6-0.6] |  | 0.8 | [0.8-0.8] |
| 3 | 0.8 | [0.8-0.8] |  | 1.2 | [1.2-1.2] |  | 0.8 | [0.8-0.8] |  | 1.3 | [1.3-1.3] |
| 4 | 1.6 | [1.5-1.6] |  | 4.1 | [4.0-4.1] |  | 1.6 | [1.6-1.6] |  | 4.2 | [4.1-4.2] |
| 5 | 4.9 | [4.9-4.9] |  | 22.0 | [21.9-22.6] |  | 4.9 | [4.9-5.0] |  | 22.6 | [22.0-23.0] |
| 6 | 28.8 | [28.1-29.0] |  | 145.0 | [142.1-145.0] |  | 29.0 | [28.2-29.1] |  | 145.0 | [144.7-145.0] |
| 7 | 153.8 | [152.0-153.9] |  | *na* | *na* |  | 153.8 | [152.0-153.9] |  | *na* | *na* |
| 8 | *na* | *na* |  | *na* | *na* |  | *na* | *na* |  | *na* | *na* |
| 9 | *na* | *na* |  | *na* | *na* |  | *na* | *na* |  | *na* | *na* |
| 10 | *na* | *na* |  | *na* | *na* |  | *na* | *na* |  | *na* | *na* |
| 15 | *na* | *na* |  | *na* | *na* |  | *na* | *na* |  | *na* | *na* |
| 20 | *na* | *na* |  | *na* | *na* |  | *na* | *na* |  | *na* | *na* |
| *Winter* |  |  |  |  |  |  |  |  |  |  |  |
| 1 | 0.5 | [0.5-0.5] |  | 0.8 | [0.7-0.8] |  | 0.3 | [0.3-0.3] |  | 0.6 | [0.6-0.6] |
| 2 | 0.8 | [0.8-0.8] |  | 1.0 | [1.0-1.0] |  | 0.6 | [0.6-0.6] |  | 0.8 | [0.8-0.8] |
| 3 | 1.3 | [1.2-1.3] |  | 3.7 | [3.7-3.8] |  | 0.8 | [0.8-0.8] |  | 1.7 | [1.7-1.7] |
| 4 | 4.7 | [4.6-4.8] |  | 19.1 | [18.9-19.9] |  | 1.8 | [1.8-1.8] |  | 5.8 | [5.7-5.8] |
| 5 | 27.0 | [25.2-28.3] |  | 136.2 | [135.0-145.0] |  | 7.3 | [7.1-7.8] |  | 33.0 | [32.9-33.3] |
| 6 | 153.8 | [153.8-155.8] |  | *na* | *na* |  | 37.8 | [37.0-37.9] |  | 156.2 | [156.2-156.2] |
| 7 | *na* | *na* |  | *na* | *na* |  | 192.1 | [188.1-192.1] |  | *na* | *na* |
| 8 | *na* | *na* |  | *na* | *na* |  | *na* | *na* |  | *na* | *na* |
| 9 | *na* | *na* |  | *na* | *na* |  | *na* | *na* |  | *na* | *na* |
| 10 | *na* | *na* |  | *na* | *na* |  | *na* | *na* |  | *na* | *na* |
| 15 | *na* | *na* |  | *na* | *na* |  | *na* | *na* |  | *na* | *na* |
| 20 | *na* | *na* |  | *na* | *na* |  | *na* | *na* |  | *na* | *na* |
| *Spring* |  |  |  |  |  |  |  |  |  |  |  |
| 1 | 0.4 | [0.4-0.4] |  | 0.7 | [0.7-0.7] |  | 0.3 | [0.3-0.3] |  | 0.5 | [0.5-0.5] |
| 2 | 0.8 | [0.8-0.8] |  | 1.0 | [1.0-1.0] |  | 0.6 | [0.6-0.6] |  | 0.8 | [0.8-0.8] |
| 3 | 1.1 | [1.1-1.1] |  | 2.9 | [2.9-3.0] |  | 0.8 | [0.8-0.8] |  | 1.6 | [1.6-1.6] |
| 4 | 3.8 | [3.8-3.9] |  | 15.9 | [15.5-15.9] |  | 1.8 | [1.8-1.8] |  | 5.0 | [5.0-5.1] |
| 5 | 20.0 | [19.5-21.7] |  | 108.0 | [94.1-110.8] |  | 6.4 | [6.1-6.8] |  | 31.9 | [31.3-31.9] |
| 6 | 145.0 | [136.2-145.0] |  | *na* | *na* |  | 35.5 | [34.9-36.4] |  | 156.1 | [156.1-156.1] |
| 7 | *na* | *na* |  | *na* | *na* |  | 158.6 | [156.2-188.1] |  | *na* | *na* |
| 8 | *na* | *na* |  | *na* | *na* |  | *na* | *na* |  | *na* | *na* |
| 9 | *na* | *na* |  | *na* | *na* |  | *na* | *na* |  | *na* | *na* |
| 10 | *na* | *na* |  | *na* | *na* |  | *na* | *na* |  | *na* | *na* |
| 15 | *na* | *na* |  | *na* | *na* |  | *na* | *na* |  | *na* | *na* |
| 20 | *na* | *na* |  | *na* | *na* |  | *na* | *na* |  | *na* | *na* |

**REFERENCES**

Kuitunen, I., Ponkilainen, V. T., Uimonen, M. M., Eskelinen, A., & Reito, A. (2021). Testing the proportional hazards assumption in cox regression and dealing with possible non-proportionality in total joint arthroplasty research: methodological perspectives and review. *BMC Musculoskelet Disord, 22*(1), 489. doi:10.1186/s12891-021-04379-2

Schemper, M., Wakounig, S., & Heinze, G. (2009). The estimation of average hazard ratios by weighted Cox regression. *Stat Med, 28*(19), 2473-2489. doi:10.1002/sim.3623

Sitlani, C. M., Lumley, T., McKnight, B., Rice, K. M., Olson, N. C., Doyle, M. F., Huber, S. A., Tracy, R. P., Psaty, B. M., & Delaney, J. A. C. (2020). Incorporating sampling weights into robust estimation of Cox proportional hazards regression model, with illustration in the Multi-Ethnic Study of Atherosclerosis. *BMC Med Res Methodol, 20*(1), 62. doi:10.1186/s12874-020-00945-9
